# Extracellular vesicles from HTLV-1 infected cells modulate target cells and viral spread

**DOI:** 10.1186/s12977-021-00550-8

**Published:** 2021-02-23

**Authors:** Daniel O. Pinto, Sarah Al Sharif, Gifty Mensah, Maria Cowen, Pooja Khatkar, James Erickson, Heather Branscome, Thomas Lattanze, Catherine DeMarino, Farhang Alem, Ruben Magni, Weidong Zhou, Sandrine Alais, Hélène Dutartre, Nazira El-Hage, Renaud Mahieux, Lance A. Liotta, Fatah Kashanchi

**Affiliations:** 1 Laboratory of Molecular Virology, School of Systems Biology, George Mason University, Manassas, VA USA; 2 Center for Applied Proteomics and Molecular Medicine, George Mason University, Manassas, VA USA; 3 International Center for Research in Infectiology, Retroviral Oncogenesis Laboratory, INSERM U1111-Université Claude Bernard Lyon 1, CNRS, UMR5308, Ecole Normale Supérieure de Lyon, Université de Lyon, Fondation Pour La Recherche Médicale, Labex Ecofect, Lyon, France; 4 Department of Immunology and Nanomedicine, Herbert Wertheim College of Medicine, Florida International University, Miami, FL USA

## Abstract

**Background:**

The Human T-cell Lymphotropic Virus Type-1 (HTLV-1) is a blood-borne pathogen and etiological agent of Adult T-cell Leukemia/Lymphoma (ATLL) and HTLV-1 Associated Myelopathy/Tropical Spastic Paraparesis (HAM/TSP). HTLV-1 has currently infected up to 10 million globally with highly endemic areas in Japan, Africa, the Caribbean and South America. We have previously shown that Extracellular Vesicles (EVs) enhance HTLV-1 transmission by promoting cell–cell contact.

**Results:**

Here, we separated EVs into subpopulations using differential ultracentrifugation (DUC) at speeds of 2 k (2000×*g*), 10 k (10,000×*g*), and 100 k (100,000×*g*) from infected cell supernatants. Proteomic analysis revealed that EVs contain the highest viral/host protein abundance in the 2 k subpopulation (2 k > 10 k > 100 k). The 2 k and 10 k populations contained viral proteins (i.e., p19 and Tax), and autophagy proteins (i.e., LC3 and p62) suggesting presence of autophagosomes as well as core histones. Interestingly, the use of 2 k EVs in an angiogenesis assay (mesenchymal stem cells + endothelial cells) caused deterioration of vascular-like-tubules. Cells commonly associated with the neurovascular unit (i.e., astrocytes, neurons, and macrophages) in the blood–brain barrier (BBB) showed that HTLV-1 EVs may induce expression of cytokines involved in migration (i.e., IL-8; 100 k > 2 k > 10 k) from astrocytes and monocyte-derived macrophages (i.e., IL-8; 2 k > 10 k). Finally, we found that EVs were able to promote cell–cell contact and viral transmission in monocytic cell-derived dendritic cell. The EVs from both 2 k and 10 k increased HTLV-1 spread in a humanized mouse model, as evidenced by an increase in proviral DNA and RNA in the Blood, Lymph Node, and Spleen.

**Conclusions:**

Altogether, these data suggest that various EV subpopulations induce cytokine expression, tissue damage, and viral spread.

## Background

The Human T-cell Lymphotropic Virus Type-1 (HTLV-1) is a bloodborne human retrovirus from the genus *Deltaretrovirus* [1]. HTLV-1 infects 10 million globally with pockets of high endemicity in the Caribbean, South America, Western and Southern Africa, Iran, Japan, and Australia [2]. HTLV-1 was first found in 1979 and published in 1980 [3, 4] and is the causative agent of an aggressive cancer known as Adult T-cell Leukemia/Lymphoma (ATLL) [5–7]. HTLV-1 also causes a set of progressive neuroinflammatory conditions known as HTLV-1-associated Myelopathy/Tropical Spastic Paraparesis (HAM/TSP) [8, 9]. HTLV-1 may cause ATLL in 3–5% of the infected population [7, 10–14], while causing HAM/TSP in 0.25–3.8% [15–17]. More recently, reports of 33.6% prevalence in a cohort of 1889 indigenous Australian patients [18] and the lack of global awareness about HTLV-1 are cause for public health concern [19]. Despite the increased efforts to understand HTLV-1 pathogenicity, more research is needed to further understand how HTLV-1 persists, evades immunosurveillance, and proliferates in the central nervous system (CNS) and other organs throughout the body.

The mode of HTLV-1 transmission, in contrast to that of the human immunodeficiency virus type-1 (HIV-1), is primarily via cell-to-cell contact involving mechanisms such as virological synapse (VS), viral biofilm (VB), cellular conduits (CCs) or tunneling nanotubes (TNTs) [20–23]. While cell-to-cell contact may limit HTLV-1 transmission rates, it also may allow the virus to remain undetected due to low numbers of free virions shown to be unstable and poorly infectious in monocytes, T-cells, and monocyte-derived dendritic cells [24–28]. The mechanisms of cell-to-cell transmission may involve peripheral and integral proteins which typically enable cellular communication and potentially viral transmission. For instance, the cluster of differentiation glycoproteins, CD45 and CD43, involved in migration, T-cell receptor signaling, and apoptosis [29], have been reported to be upregulated in HTLV-1 infection and may play a role in VB formation [30] and viral transmission [31]. Similarly, the adhesion molecules ICAM-1 and LFA-1 have been shown to participate in VS transmission [21, 32–34]. Interestingly, our recent data has shown that these cellular proteins are upregulated not only in the HTLV-1 infected HUT102 cell line, but also in small membrane-bound structures, known as Extracellular Vesicles (EVs), as they are secreted by infected cells and contain CD45, CD43, ICAM-1 and LFA-1 [35].

EVs have been shown to play important roles in modulating processes such as cell signaling, cell dysfunction, inflammation, angiogenesis, and viral activation, among others [35–43]. We have recently shown that EVs secreted from HTLV-1 infected cells (HTLV-1 EVs) increase cell-to-cell contact in uninfected T-cells and subsequently facilitate more efficient viral transmission both in vitro and in vivo [35]. EVs have complex biogenesis pathways classified as “conventional” and “unconventional” pathways [38]. A “conventional” pathway may be through the endosomal sorting complex required for transport (ESCRT) pathway which utilizes tetraspanins (i.e., CD63, CD81, and CD9) and accessory proteins (i.e., TSG101, VPS4, and others) to incorporate cargo (such as RNA and proteins) into vesicles generated from invagination of segments of the plasma membrane (endosomes) into a membrane-bound structure known as the multi-vesicular body (MBV). This pathway may be used by cells as a degradative pathway for ubiquitinated cargo. In the MBV, the cargo is packaged into intraluminal vesicles (ILVs), which upon fusion of the MBV with the plasma membrane may be released as exosomes into the extracellular environment. On the other hand, “unconventional” EV biogenesis pathways may include secretory autophagy, involving degradative pathways triggered by cellular stress (i.e., starvation, DNA damage, and cytotoxicity). Autophagy is an essential cellular function that ensures homeostasis and prevents accumulation of cellular waste. Many viruses, including HTLV-1, have been shown to highjack autophagy via viral proteins [44–51]. This may then render autophagy deficient by preventing formation of the autolysosome, a membrane-bound structure containing enzymes that digest cellular waste. The HTLV-1 protein Tax has been reported to inhibit formation of the autolysosome by blocking fusion of the lysosome (vesicle containing degradative enzymes) with the amphisome (vesicle containing cargo targeted for degradation) [38, 50, 52]. Therefore, understanding which EV biogenesis pathway yields a specific EV population may be essential to preventing some of the EV-mediated effects observed in HTLV-1 infection.

We have previously shown extensive isolation and separation of EVs away from viruses [35–37, 39, 42, 53–57] and recently shown that EVs from HTLV-1, as well as HIV-1, infected cells may be separated away from virus using iodixanol density gradients [35, 42]. An alternative method for EV isolation is via differential ultracentrifugation (DUC) into three major populations, 2 k EVs (2000×*g* centrifugation), 10 k EVs (10,000×*g* centrifugation), and 100 k EVs (100,000×*g* centrifugation) [58–60]. Additionally, Nanotrap nanoparticles may be used to enrich for EVs or virions for use in downstream assays, such as Western Blot and RT-qPCR [61].

HTLV-1 infection can be associated with a robust hijacking of the host's cellular machinery, including the utilization of the ubiquitous secretion pathways involving EVs [35]. Our data showed that HTLV-1 infected cells may secrete viral proteins packaged into EVs and that an iodixanol density gradient allowed the separation of EVs into fractions in three major complexes (low-, mid-, and high-density fractions) [35]. Low-density fractions carried EVs associated with functional viral proteins, such as Tax; structural proteins, such as gp46; and unprocessed viral proteins, such as gp61. In mid-density fractions, gp61 and the matrix protein, p19, were almost exclusively packaged, with trace levels of Tax and gp46. However, high-density fractions were devoid of gp61 and contained abundant p19, gp46, Tax, suggestive of the presence of mature virions mixed with large or densely packed EVs. It is possible that these EVs are also associated with mature viral proteins, resembling a virion. Although high-density fractions contained all the prerequisites of an HTLV-1 virion, these were still not infectious [35]. Here, we used Nanotrap particles to enrich for EVs in isolates from DUC (2 k, 10 k, and 100 k EVs) that correlate with high-density, mid-density, and low-density fractions, respectively.

In this manuscript, we have attempted to better understand various EV populations separated by differential ultracentrifugation to modulate recipient cell cytokine production and increase viral spread (in vitro and in vivo). The modulation of cytokine production in specific cell types may promote the development of CNS inflammation, potentially related to HAM/TSP. Additionally, cargo in specific EV populations may promote the development of inflammation and viral spread in organs and peripheral blood, potentially contributing to the development of ATLL. Overall, we found that HTLV-1 EVs have the potential to regulate cell migration, inflammation, angiogenesis, and tissue damage. Further mechanistic understanding of these EVs may allow the development of therapeutics against viral transmission and development of neurodegeneration in HAM/TSP patients.

## Results

### Viral proteins are differentially packaged into EVs from HTLV-1 infected cells

In this manuscript we sought to further investigate HTLV-1 EVs via the use of DUC, generating three distinct EV populations: 2 k, 10 k, and 100 k EVs. The advantage of utilizing DUC exclusively over iodixanol gradients is that DUC can result in a higher EV yield and does not require additional reagents that may interfere with downstream assays. We expected 2 k, 10 k, and 100 k EVs to resemble EVs sedimented in high-, mid-, and low-density Iodixanol fractions, respectively. Using Nanoparticle Tracking Analysis (NTA), we observed that the average diameter of 2 k EVs were about 189 nm, 10 k around 171 nm, and 100 k around 129 nm (Additional file 1: Fig. S1). These data support the hypothesis that 2 k EVs are denser than 10 k and 100 k EVs, also resembling EVs that would sediment in high-density fractions, as shown in our previous publication [35].

**Fig. 1:**
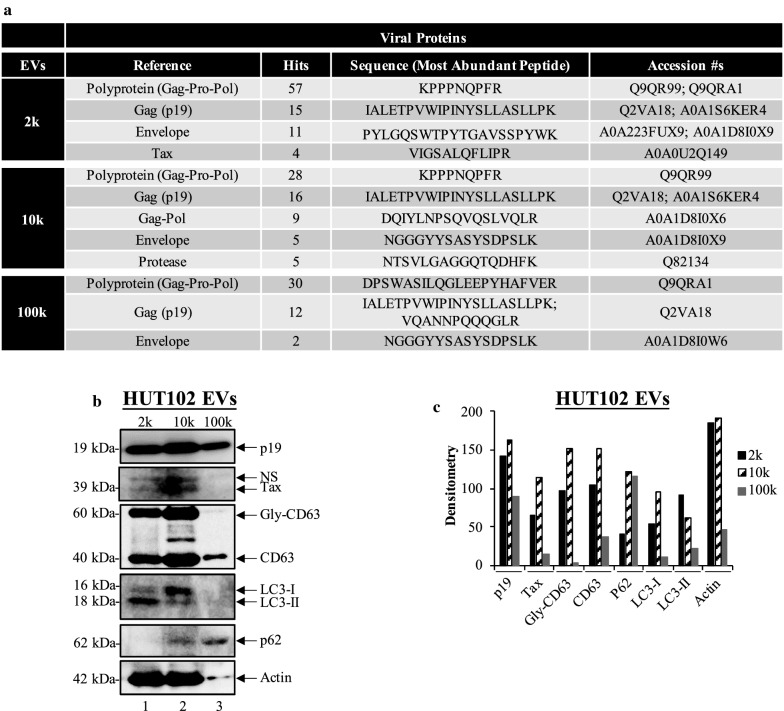
Tax and p19 viral proteins are present in EV populations derived from HTLV-1 infected cells. **a** Viral protein distribution was evaluated in different EV populations (i.e., 2 k, 10 k, and 100 k) derived from HUT102 cells. Mass spectrometry data was searched against a fully tryptic HTLV-1 database (UniProt) via Proteome Discoverer 2.1. **b** Different EV populations (2 k, 10 k, and 100 k) of HUT102 were obtained via differential ultracentrifugation and 30 μg was loaded into each lane. Viral proteins (p19 and Tax), EV marker (CD63), autophagy proteins (LC3-I, LC3-II, and p62) and Actin were detected using Western blot. **c** Quantification of Western blot bands by densitometry analysis

EVs play an important role in cell-to-cell communication, with potential roles in autocrine, paracrine, and endocrine signaling [35, 41, 62–66]. EV biogenesis may be connected to normal cellular processes, such as autophagy, by serving as intracellular “vehicles” that carry cargo targeted for degradation [38, 40, 67–69]. Additionally, we have shown that infection with viruses, such as HTLV-1 and HIV-1, cause the release of EVs with viral cargo (i.e., viral protein and non-coding RNA). In order to further understand the capacity of EVs to carry viral and human proteins, we performed proteomic analysis of the 2 k, 10 k, and 100 k EVs from HTLV-1 infected cells. EV samples were reduced by dithiotreitol, alkylated with iodoacetamide, trypsinised overnight, desalted using C-18 ZipTips, dried under nitrogen, and reconstituted with Formic Acid prior to injection into the Liquid Chromatography Tandem Mass Spectrometry (LCMS/MS) instrument. Acquired MS/MS spectra were searched against a fully tryptic HTLV-1 database (UniProt) with Proteome Discoverer 2.1. Data in Fig. 1a show the proteomic distribution of viral proteins. To qualitatively analyze the viral proteomic data, we evaluated the relative abundance of each protein present (“ + ”) within an EV type. The relative abundance of a protein was then indicated by “ + ” if the peptide hits were detected between 1 to 5 times, “ +  + ” between 6 to 15 times, “ +  +  + ” between 16 to 29 times, and “ +  +  +  + ” for 30 times and above. In 2 k EVs, the precursor polyprotein Gag-Pro-Pol (most frequent), Gag (p19), Envelope, and Tax proteins were detected (Gag-Pro-Pol ^++++^  > Gag^++^  > Envelope^++^  > Tax^+^). In 10 k EVs, Gag-Pro-Pol (most frequent), Gag (p19), Gag-Pol, Envelope, and Protease were detected (Gag-Pro-Pol ^+++^  > Gag^+++^  > Gag-Pol^++^  > Envelope^+^  > Protease^+^). In 100 k EVs, Gag-Pro-Pol (most frequent), Gag, and Envelope were detected (Gag-Pro-Pol ^++++^  > Gag^++^  > Envelope^+^). All EVs contained Gag-Pro-Pol, Gag and Envelope related proteins. Unique viral proteins were detected in 2 k (i.e., Tax), 10 k (i.e., Protease), while 100 k EVs contained no unique viral proteins. Overall, this data suggests potential differential packaging of viral proteins into different EV types.

To further validate the presence of viral proteins in EVs we inspected EVs from two different HTLV-1 infected cell lines, HUT102 and ATL-16. Data in Fig. 1b show Western blot of EVs separated by DUC and observed that the Gag related matrix protein, p19 was present in 2 k, 10 k, and 100 k EVs from both HUT102 (lanes 1–3) and ATL-16 (Additional file 1: Fig. S2). Densitometry analysis confirmed higher abundance of p19 in the 10 k EVs from HUT102 (Fig. 1c). Next, we observed presence of Tax in 2 k, and more so, in 10 k EV from HUT102 cells. It is important to mention that Tax was also detected in the MS analysis, however it was present as a peptide fused to Gag (13 hits; see Additional file 2) and therefore considered as not significant. Detection of Tax in WB serves as a confirmation. In the case of EVs from ATL-16 cells, Tax also was predominantly present in the 10 k subpopulation. We also assayed for presence of the common EV marker, CD63, and observed different patterns of protein modification. In the case of HUT102, the 2 k and 10 k EVs contained glycosylated and unmodified CD63, however the 100 k EVs contained almost exclusively unmodified CD63. On the other hand, EVs from ATL-16 cells contained primarily glycosylated CD63, with exception of the 10 k EV subpopulation, which also contained unmodified CD63 (Additional file 1: Fig. S2). We have previously observed the existence of particular patterns of CD63 modifications. EVs isolated from HAM/TSP patient PBMCs were positive for both the glycosylated and unmodified CD63 populations, whereas those isolated from normal PBMC donors only contained unmodified CD63 [41]. Overall, our data suggests that both the 2 k and 10 k EVs from HTLV-1 infected cells have the potential to modulate cell responses conducive of disease progression, due to the detection of viral proteins (Gag/p19 and Tax) and of EV markers previously associated with advanced stages of HTLV-1 infection ((glycosylated (Gly-CD63) and unmodified CD63)). However, the 100 k EV population has unique and previously unseen characteristic in the context of HTLV-1, suggestive of a vesicle with the ability to interact with cells compared to 2 k/10 k EVs, but without the oncogenic effects of Tax.

Next, we investigated for the presence of autophagy proteins, as these may be suggestive of the biogenesis pathway generating each EV population. In Fig. 1b, we also probed for LC3-I/II, an important protein in the formation of the autophagosome, a membrane-bound structure responsible for encapsulating intracellular components. LC3-I elongates the pre-autophagosome’s membrane and promotes engulfment of components in a ubiquitin-like mechanism of targeting. Once the autophagosome is formed, LC3-II is normally detected, suggesting flux from LC3-I to LC3-II and advanced stages of autophagy, which could include degradation of cargo and secretion of vesicles [38, 70]. Therefore, LC3-I in the extracellular environment could be suggestive of high levels of cytoplasmic LC3-I resulting in secretion in EVs. However, LC3-II alone in the extracellular environment could indicate the secretion of vesicles derived from mature stages of autophagy. The autophagy protein p62, could be used to evaluate autophagy flux since it is essential to the activation of autophagy. Upon the p62-mediated recruitment of autophagy proteins and formation of mature autophagosomes, p62 levels decrease due to potential degradation [71].

Interestingly, our Western blot data revealed increased presence of LC3-II marker in 2 k EVs from HUT102 cells, but not in ATL-16 (Additional file 1: Fig. S2). In contrast, observations of 10 k EVs from HUT102 cells revealed increased LC3-I levels, whereas ATL-16 contained mostly LC3-II. In order to validate our observations, we probed for the p62 protein. Specific cytoplasmic targets, such as viral components (among others), may stimulate p62 via pattern recognition receptors (PRRs), initiating recruitment of autophagic machinery [38, 70, 72]. Activation of autophagy requires initial upregulation of p62; however, upon formation of the autophagosome, decreased p62 levels have been reported [70]. Here we found that HUT102/ATL-16 2 k EV did not contain p62, however both 10 k and 100 k (more abundant) contained p62.

Overall, 2 k EVs contained mature markers of autophagy as evidenced by presence of LC3-II and no p62, whereas 10 k and 100 k EVs contained markers of pre-autophagosomes (LC3-I and p62; early stages of autophagy). This data is suggestive that 2 k EVs may carry viral components that have undergone some degradation. On the other hand, 10 k EVs may potentially contain viral proteins targeted for autophagic degradation that may have been interrupted, resulting in secretion through the EV pathway.

### Proteomic analysis of human proteins in EVs from infected cells

**Fig. 2:**
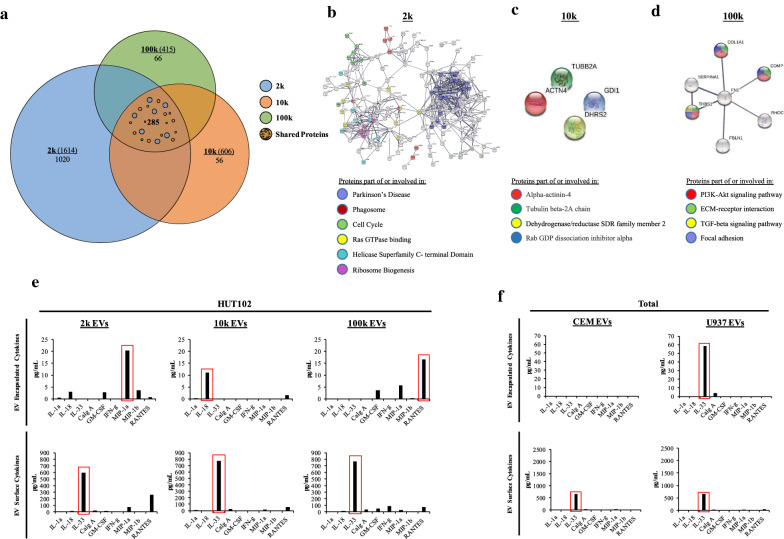
Proteomic analysis of Human proteins in HTLV-1 EVs. **a** Venn diagram of 2 k, 10 k, and 100 k EVs showing total number of proteins identified (in parenthesis) and unique proteins (underneath total proteins). Mass spectrometry data was searched against fully tryptic indexed Homo sapiens database (UniProt) via Proteome Discoverer 2.1. Next, Protein–Protein Interaction (PPI) network was evaluated for unique proteins in **b** 2 k, **c** 10 k, and **d** 100 k EVs. Predicted PPIs were analyzed by STRING to determine the pathway interaction of proteins in each EV. PPIs were analyzed from STRING to determine the pathway enriched in each EV population. In the PPI network, nodes represent Proteins (circles), Edges represent Protein–Protein Associations (lines), and color represent related pathway or family of protein (colored circles). High confidence levels of 0.7–0.9 were used for this analysis. **e** HTLV-1 EVs (2 k, 10 k, and 100 k) and **f ** control EVs (CEM and U937) were evaluated using a Luminex platform for presence of encapsulated or surface-bound cytokines. Only 9 representative EVs were selected out of a panel of 33 cytokines. Red boxes show the most abundant cytokines across the different EV populations

We next analyzed presence of human proteins in each EV population (2 k, 10 k, and 100 k EVs) via mass spectrometry. EV samples were processed as indicated previously for analysis of viral proteins, however acquired MS/MS spectra were searched against a fully tryptic indexed Homo sapiens database (UniProt) with Proteome Discoverer 2.1. The 2 k population contained 1,020 unique proteins of interest out of a total 1,614 proteins; 10 k population contained 56 unique proteins out of 606; and 100 k population contained 66 unique proteins out of 415 total proteins (Fig. 2a). Therefore, the 2 k population contains the highest variety of human proteins (2 k > 10 k > 100 k).

Next, we examined whether 2 k, 10 k, and 100 k EVs contained unique human proteins with roles in processes related to HTLV-1 pathogenesis. Therefore, we analyzed for functional protein interactions and generated a Protein–Protein Interaction (PPI) network using the Search Tool for the Retrieval of Interacting Genes (STRING) to predict relevant functional interactions [73]. The PPI network of 2 k EVs was enriched in proteins involved in Parkinson’s Disease, Phagosome formation, Cell Cycle, Ras GTPase, Helicase Superfamily Terminal Domains, and Ribosome Biogenesis (Fig. 2b). The 10 k EVs contained noninteractive proteins belonging to the family of Alpha-Actinin-4; Tubulin beta-2A chain; Dehydrogenase/reductase SDR family member 2; and Rab GDP dissociation inhibitor alpha family proteins (Fig. 2c). Finally, the 100 k EVs contained a PPI network interconnected by fibronection-1 (FN1) with proteins involved in PI3K-Akt signaling, ECM-receptor interaction; TGF-beta signaling, and Focal adhesion with a high degree of confidence (> 0.7) (Fig. 2d). Overall, the 2 k population contains several proteins with interconnected potential functions in disease (i.e., Parkinson’s) and in regulation of cell cycle, autophagy, euchromatin and gene activation, and protein synthesis/translation, and the 100 k EV proteins were all connected to FN1, involved in the support of cell migration, cell differentiation, and tissue repair [74]. The 10 k EVs contained noninteractive proteins with minimal complementary effects.

Finally, it has already been reported that EVs can modulate immune responses by promoting expression of cytokines [75], or of proteins important for immune cell interaction, such as upregulation of ICAM-1 [35, 76]. More importantly, EVs have the potential to carry cytokines (encapsulated or membrane-bound) and molecules, such as ICAM-1, denoting the importance EVs in pathogenesis [35, 75]. Additionally, the cytokine profile in EVs has been shown to be consistent with the type of cell or organ where it originates [77]. Using high throughput ELISA, we screened for cytokines encapsulated or present on the surface of EVs (2 k,10 k, and 100 k). Data in Fig. 2e show that the 2 k EVs encapsulated high-levels of the proinflammatory cytokine involved in macrophage activation, known as Macrophage Inflammatory Proteins (MIP-1a). On the other hand, 10 k EVs encapsulated the cell proliferation cytokine IL-18, and 100 k EVs encapsulated the chemokine RANTES (RANTES: regulated upon activation normally T-cell expressed and secreted). However, on the surface of EVs the predominant cytokine was IL-33, known for regulating gene expression and promoting secretion of IL-4, IL-5, IL-9 by T-helper, mast, eosinophils, and basophils cells [78, 79]. IL-33 was on the surface of all three EV populations (2 k, 10 k, and 100 k EVs). EVs from uninfected T-cells (CEM) and myeloids (U937) were also evaluated, and cytokine levels were low compared to HUT102 EVs, except for IL-33 (Fig. 2f). Overall, this suggests that EVs from HTLV-1 infected cells contain higher cytokine levels than EVs from uninfected cells, and that the levels of inflammatory molecules vary in or on the surface of EVs according to the EV subpopulation.

### Functional effects of 2 k, 10 k and 100 k HTLV-1 EVs on uninfected recipient T-cells

**Fig. 3:**
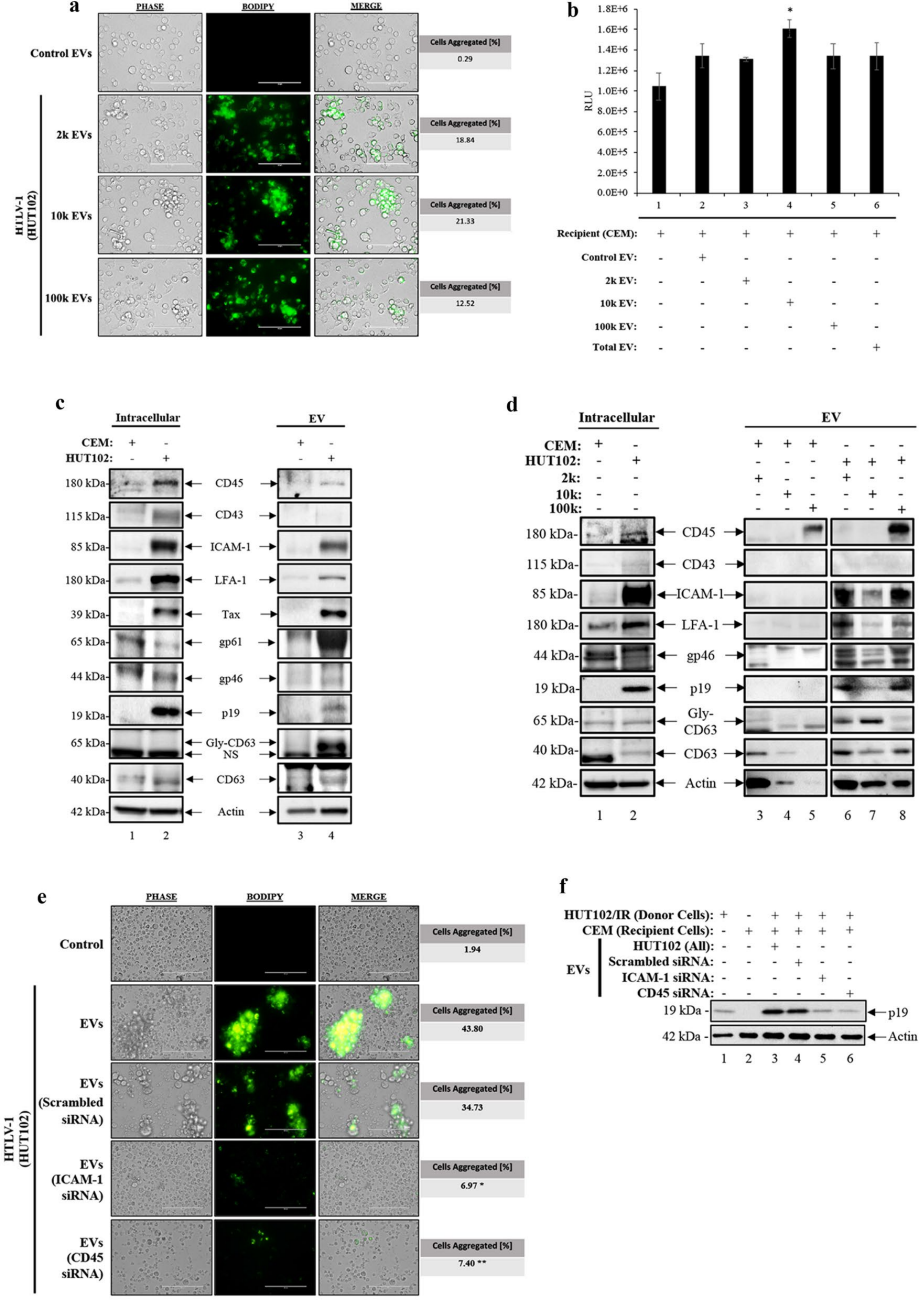
EV subpopulations enhance cell-to-cell contact, which is abolished by ICAM-1 or CD45 siRNA. **a** Fluorescent Microscopy was used to track BODIPY™ 493/503 labeled EVs (2 k at 1: 6,200 cell to EV; 10 k at 1: 20,000; 100 k at 1: 8,200, and CEM at 1: 8,866) added to CEM recipient cells. Analysis was performed 24 h post-treatment and quantified total and aggregated cells. Scale bar (100 μM). **b** Cell viability of recipient cells (CEM; 5 × 10^5^ cells/mL, in biological triplicates) incubated with HTLV-1 2 k, 10 k, 100 k, and total EV populations (CEM and HUT102). **c** Cells and corresponding total EVs (CEM and HUT102), **d** as well as subpopulations (2 k, 10 k and 100 k EVs), were analyzed via Western blot for cell surface proteins (CD45, CD43, ICAM-1, LFA-1), HTLV-1 proteins (gp46, and p19), EVs (CD63), and Actin. **e** The 10 k and 100 k EVs were isolated (single 100,000 × *g* spin) from HUT102 cells transfected cells with siRNA against ICAM-1, CD45, or scramble (for 3 days). All samples were labeled with BODIPY and then incubated with CEM cells at a ratio of 1:10,000 cell to EV for 3 days. Total HUT102 EVs was used as positive control for enhanced cell-to-cell contact and CEM cells alone or with scrambled siRNA as a negative control. The images were taken by Fluorescent Microscopy. **f** HTLV-1 donor cells (HUT102) were irradiated (IR; 10 Gy) to induce cell cycle arrest and treat recipient cells (CEM) in fresh exo-free media for 4 days. Western blot analysis was performed for p19 and Actin. Statistical analyses were performed using two-tailed Student’s t test, “*” for p ≤ 0.05 and “**” for p ≤ 0.01

We have recently shown that HUT102 EVs (HTLV-1 EVs) promote cell-to-cell contact between uninfected T-cells [35]. Here, we attempted to elucidate whether a particular HTLV-1 EV subtype (i.e., 2 k, 10 k, and 100 k) is responsible for the increased cell-to-cell contact previously observed. Various size EVs were labeled with BODIPY and used to treat uninfected CEM T-cells. Using fluorescent microscopy, we observed uptake of all size EVs 24-h post-treatment (Fig. 3a). All HTLV-1 EV subtypes promoted increased aggregation of CEM cells, with the largest agglutination with 10 k (21.33%), followed by 2 k (18.84%), and least with 100 k (12.52%) EVs (10 k > 2 k > 100 k EVs). Finally, CEM cell viability was unaffected by HTLV-1 EV treatment, with only a slight but significant increase when treated with 10 k HTLV-1 EVs (Fig. 3b; lane 4). Furthermore, this data suggests that HTLV-1 EVs from 2 k, 10 k, and 100 k populations have the potential to increase agglutination and cell-to-cell contact without causing cell death. Altogether, this data suggests that the increase in agglutination and cell-to-cell contact is a consequence of treatment with EVs, and not an artifact related to treatment and cell death.

HTLV-1 EVs from all three populations ubiquitously promoted increased cell-to-cell contact, with potential increased potency in 10 k EVs. Cell-to-cell contact may be mediated by cell surface proteins and adhesion molecules such as CD45, CD43, ICAM-1, and LFA-1 [21, 22]. We have recently shown that EVs from HTLV-1 infected cells (with and without transcriptional activation via irradiation) contain protein markers involved in mechanisms of viral transmission. More specifically, we have shown that HTLV-1 EVs contain proteins involved in the formation of VB (i.e., CD45) and VS (i.e., ICAM-1) and that neutralizing antibodies against these two proteins may decrease the agglutination and viral spread in cell lines and PBMCs [35].

Here we sought to further investigate expression levels of proteins involved in HTLV-1 transmission into recipient uninfected cells (CEM; intracellular proteins), EVs from uninfected cells (CEM EVs; EV-associated proteins), infected donor cells (HUT102 cells; intracellular proteins), and EVs from HUT102 cells (HTLV-1 EVs; EV-associated proteins). When using Western blot analysis, intracellular protein expression levels of CD45, CD43 (binding partner of CD45), ICAM-1, and LFA-1 (binding partner of ICAM-1) were significantly elevated in HTLV-1 infected HUT102 cells (Fig. 3c; lane 2) compared to uninfected CEM cells (Fig. 3c; lane 1). Intracellular viral proteins were also probed as controls for infection, with bands for Tax, gp61, gp46, and p19 present only in the intracellular material from HUT102 cells (Fig. 3c; lanes 2). Moreover, the tetraspanin and EV marker, CD63 [80], showed equal protein expression levels between HUT102 and CEM cells. We next investigated the EV-associated protein levels in HTLV-1 EVs and CEM EVs. Cell protein markers and adhesion molecules present in VBs (CD45 only) or involved with the formation of VS (ICAM-1 and LFA-1) were almost exclusively present in HTLV-1 EVs (Fig. 3c; lane 4), with traces of CD45 and LFA-1 present in CEM EVs (Fig. 3c; lane 3). Not surprisingly, viral proteins Tax, gp61, and p19 were present only in HTLV-1 EVs, as previously observed [35]. However, an interesting observation was made regarding an EV marker protein, where the unmodified band for CD63 was expressed in both HTLV-1 and CEM EVs, but the modified, glycosylated form of CD63 (Gly-CD63) was only observed in HTLV-1 EVs (Fig. 3c; lane 4 vs. lane 3).

Separation of HTLV-1 EVs (2 k,10 k, and 100 k) would allow further characterization of which EV population is responsible for promoting cell-to-cell contact and viral spread. Data in Fig. 3d shows that CD45 was abundant in 100 k EVs from CEM and HUT102 (lanes 5 and 8). However, CD43 was not seen in EVs obtained from both cell lines (lanes 3–8). EVs from infected cells had high expression of ICAM-1 in 2 k and 100 k (lanes 6 and 8) and less in 10 k EVs (lane 7). Control EVs (CEM EVs) did not contain detectable ICAM-1 (lanes 3–5). Similarly, LFA-1 was detected in 2 k, 10 k, and 100 k EVs, but with reduced signal in 10 k EVs and no detectable signal in Control EVs. We also assessed for presence of viral proteins and observed their presence only in HTLV-1 EVs. We observed increased expression of gp46 and p19 in 2 k and 100 k (lanes 6 & 8) compared to 10 K (lane 7). Finally, Gly-CD63, typically found in EVs from infected cells ((see [41, 54]), was observed primarily in 2 k and 10 k, but not in 100 k EVs or in Control EVs.

Altogether, these data suggest that HTLV-1 infected cells have an upregulated expression of proteins important for viral transmission via mechanisms of VB (CD45 and CD43) and VS (ICAM-1 and LFA-1) which are also overexpressed extracellularly on HTLV-1 EVs in the case of CD45, ICAM-1 and LFA-1. Furthermore, the presence of HTLV-1 EVs and their cargo may promote cell-to-cell contact and subsequent viral transmission. HTLV-1 EVs may potentially adhere and "decorate" uninfected cell surfaces, facilitating agglutination without compromising cell viability.

### Inhibition of ICAM-1 and CD45 via small interfering RNA prevents packaging into HTLV-1 EVs and cell-to-cell contact

We have shown that the mechanisms of cell-to-cell contact in HTLV-1 infection may be mediated by EVs, especially during enhanced viral spread [35]. More specifically, we have shown that EVs from infected cells carry proteins known to be involved in cell-to-cell contact (ICAM-1 and CD45) and that neutralization with antibodies protected cells from enhanced viral spread [35]. Here, we attempted to go one step further and validated these observations by inhibiting translation of ICAM-1 and CD45 from the infected donor cells using small interfering RNA (siRNA). EVs from siRNA (scrambled, CD45, and ICAM-1) treated donor cells were isolated, as described previously (Fig. 3a), and protein levels evaluated using Western blot analysis (Additional file 1: Fig. S3). Addition of the scrambled siRNA (lane 1) showed baseline levels of CD45, ICAM-1, and Actin proteins in these EVs. In addition, CD45 and ICAM-1 levels were suppressed when treated siRNA against CD45 (lane 2) and siRNA against ICAM-1 (lane 3), respectively. We next used these EVs to treat recipient uninfected cells (Fig. 3e). We observed that EVs from infected cells treated with siRNA against ICAM-1 reduced cell aggregation by 6.97% and siRNA against CD45 by 7.40% (Fig. 3e). As expected, positive and negative controls showed a total aggregation at 43.80% and background at 1.94%, respectively [35]. We also examined the uninfected CEM target cells (Recipient cells) by performing a Western blot probing for p19 in the WCE of recipient cells (Fig. 3f). The p19 levels were less abundant in CEM cells treated with EVs from infected cells treated with siRNA against ICAM-1 and CD45, suggesting that inhibiting ICAM-1 or CD45 resulted in a potential decrease of viral spread into target cells. Altogether, this data suggests that the virus may use intracellular pathways to promote packaging of ICAM-1 and CD45 into EVs, that would subsequently enhance cellular aggregation and viral spread.

### Functional effects of HTLV-1 EVs on angiogenesis and inflammation

**Fig. 4:**
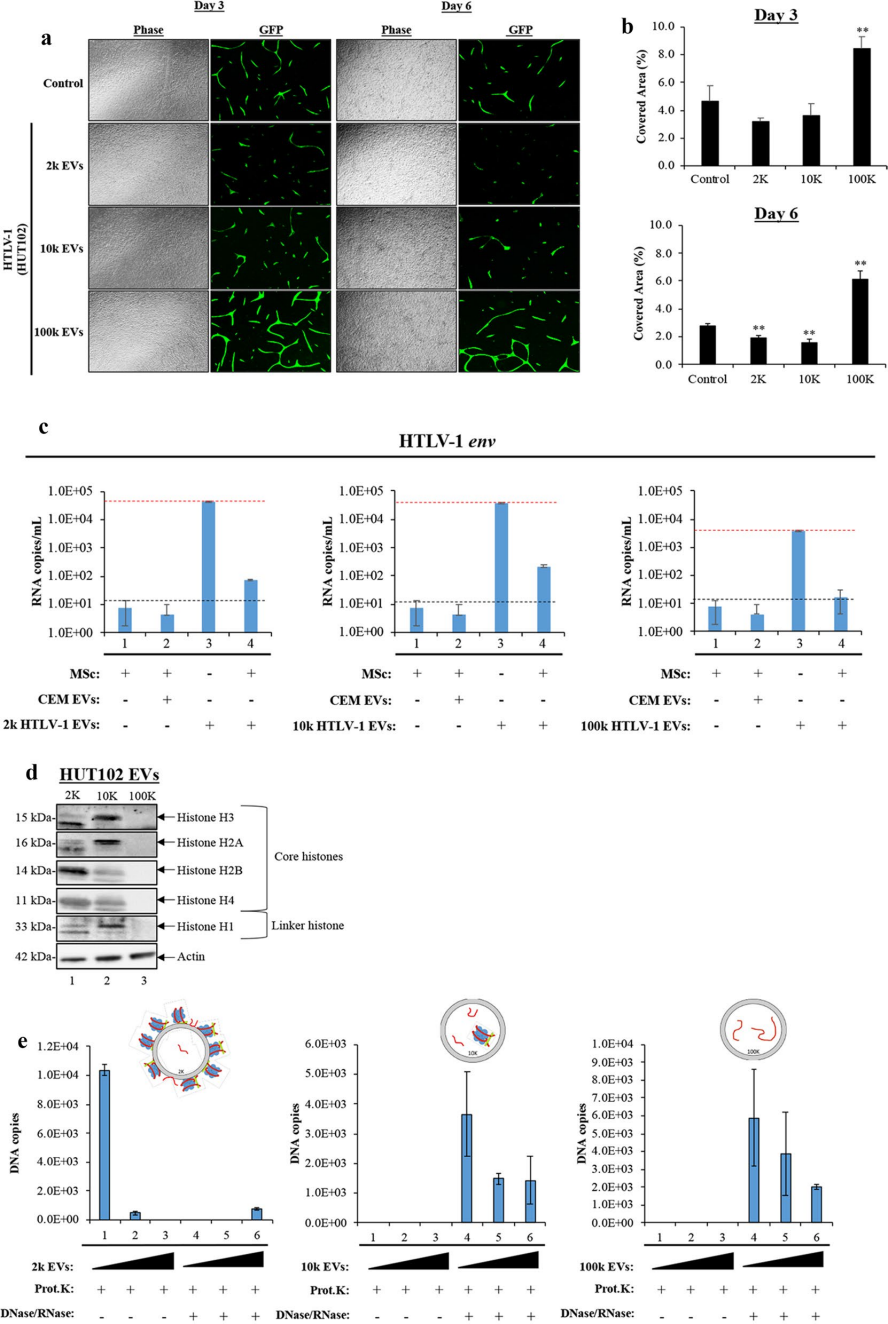
Functional Effects of HTLV-1 EVs on Angiogenesis and Inflammation. **a** An angiogenesis assay in technical and biological triplicate was used to determine the effect of distinct HTLV-1 EVs (2 k, 10 k, and 100 k) on tubular formation in a co-culture of mesenchymal stem cells (MSCs) and aortic endothelial cells (AEC). EV-treated cells (1:2000 recipient cell to EV ratio). Positive control cells received complete, undiluted medium. Additional dose of EV treatment was given to the cells at day 4. Representative images were taken at days 3 and 6 showing tubular formation in response to the indicated treatment. **b** The image processing software WIMASIS was used to calculate the percentage of area covered by tubules (n = 3) on day 3 and day 6. **c** RT-qPCR results showing *env* RNA copy numbers of mesenchymal stem cells treated with CEM EVs (control) and different populations of HTLV-1 EVs: 2 k (left panel), 10 k (middle panel), and 100 k (right panel). A set of dashed black vertical lines (---) were used to indicate baseline *env* RNA copy numbers. A set of dashed red vertical lines ( ) were used to indicate the levels of starting material, suggestive of the minimum *env* RNA copy numbers necessary for EVs to increase vial spread in MSc. **d** Western blot analysis for core histones (H3, H2A, H2B, and H4), linker histone (H1) and actin in HUT102 EVs (2 k, 10 k, and 100 k). **e** GAPDH DNA levels (representative of nucleosomes) in 2 k, 10 k, and 100 k HUT102 EVs treated with proteinase K and DNase/RNase were evaluated by was quantitated by q-PCR. A two-tailed student t-test was used to evaluate statistical significance with “**” for p-values ≤ 0.01, indicating the level of significance relative to untreated (Control) samples

We have recently used an in vitro angiogenesis assay to examine the effects of stem cell EVs on the formation of vascular structures [43]. The assay consists of a mixture of mesenchymal stem cells (MSCs) and GFP expressing human aortic endothelial cells (AECs) that were cultured together to investigate the effects of distinct HTLV-1 EV populations. The AECs in this co-culture develop vascular-like tubules, which may resemble those produced by endothelial cells in the blood–brain barrier (BBB). We utilized this system to evaluate the functional effects of 2 k, 10 k, and 100 k HTLV-1 EVs on tubular formation or deterioration. Using an angiogenesis assay, data in Fig. 4a showed differential effects of 2 k, 10 k, and 100 k HTLV-1 EVs on tubular formation. At 3 days post-treatment, 2 k and 10 k EVs showed deterioration, as evidenced by shorter and thinner tubules when compared to control (Fig. 4a; Day 3, GFP panels). In contrast, 100 k HTLV-1 EVs displayed longer tubules of consistent thickness, similar to control images. Similar results were observed at day 6 with further deterioration of tubules when using 2 k and 10 k (Fig. 4a; Day 6, GFP panels). The changes in tubule integrity were quantitated by measurement of fluorescent signal on tubules, displayed as a percentage of the total covered area (Fig. 4b). Altogether, these data suggest that 2 k and 10 k EVs have detrimental effects on cells (i.e., Mesenchymal stem cells and epithelial cells), potentially making them contribute to HTLV-1 pathogenesis, such as in the case of HAM/TSP. These negative effects could be due to the high abundance of viral proteins/RNA and human proteins in these two fractions. On the other hand, 100 k EVs may have certain protective and growth effects. Further research is needed to elucidate the mechanism of this observed protection.

HTLV-1 EVs have previously been shown to be internalized by MSCs, resulting in increased proliferation and activation of NF-κB pathway [81]. We therefore examined whether HTLV-1 EVs alone are sufficient to enter (from the angiogenesis assay) and allow viral replication in MSCs. The RT-qPCR analysis was performed on MSCs (Fig. 4c; lane 1) treated with various EVs (lanes 2–4) for 9 days. The starting 2 k, 10 k, and 100 k EV material contained *env* RNA at approximately 5 × 10^4^ copies/mL, 5 × 10^4^ copies/mL, and 5 × 10^3^ copies/mL, respectively (lane 3). However, *env* RNA levels in MSCs treated with 2 k, 10 k, and 100 k EVs showed no increase of newly synthesized RNA (lane 4). We also performed additional RT-qPCR analysis in biological triplicate to evaluate the levels of genomic RNA in EVs and observed the highest levels in 2 k and 10 k EVs (Additional file 1: Fig. S4). These data suggest that while HTLV-1 EVs may have detrimental effects on recipient cells and adjacent bystander cells (i.e., endothelial cells), it does not establish a new productive infection in these cells.

In order to further examine the potential mechanisms of cellular damage, we sought to examine for the presence of damage associated molecular patterns (DAMPs) in HTLV-1 EVs. Some of the most common DAMPs are cytoplasmic organelles and nuclear components located in the extracellular environment, normally as a consequence of disease or cellular damage, leading to proinflammatory responses [82]. More recently, DAMPs in the form of heat shock proteins, DNA, histones, and nuclear proteins, such as High Mobility Group 1 [83–85], have been found associated with EVs from cells responding to potential infection and from cancer cells [86, 87]. Interestingly, proteomic analysis revealed presence of the HMGB1 and heat shock proteins almost exclusively in the 2 k EV population, and others in the field have previously made similar observations [88]. In Fig. 4d we show a representative Western blot where the 2 k EV population (lane 1) shows strong bands for histones H3, H2A/B, and H4, with histone modifications present in all samples. The 10 k EVs (lane 2) contained high levels of histones H3, H2A, and H1, and trace levels of H2B and H4. It is important to note that 2 k and 10 k EVs also showed varying histone modifications present in H3, H2A, H2B, and H1 proteins (compare lanes 1 and 2). The 100 k EVs (lane 3) contained no detectable histones. We hypothesized that if the histones are bound to DNA, and carried by the EVs bound to their surface, then use of a proteinase K and DNase/RNase cocktail would allow digestion of DNA, RNA and proteins (histones). However, if cargo is encapsulated in EVs, then the digestion cocktail would not have effects on the EV cargo. Additionally, we used freeze/thaw to rupture EV membranes and release encapsulated cargo. In Fig. 4e using GAPDH DNA as a generic marker of nucleosome (DNA + histones), we observed most of the nucleosomes outside of the 2 k EVs (Fig. 4e; left panel), and some nucleosomes inside 10 k EVs (Fig. 4e, center panel). Interestingly, we observed presence of DNA inside the 100 k EV, however, since we could not detect histones in 100 k EVs (Fig. 4e; lane 3, right panel), we concluded that the DNA is mostly free in the 100 k EV. These results indicate differences in 2 k, 10 k, and 100 k EV composition, which may regulate angiogenesis through various DAMPs.

### HTLV-1 EVs promote differential expression of IL-8, IL-6, and RANTES in CNS related cells

**Fig. 5:**
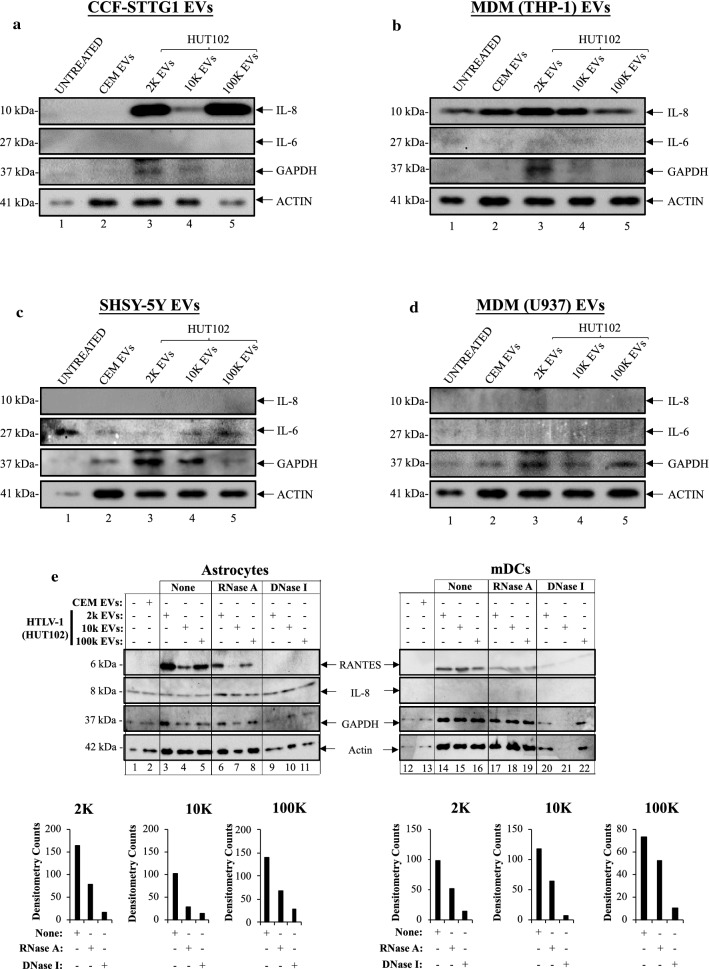
HTLV-1 EVs promote differential expression of IL-8, IL-6, and RANTES in CNS related cells. HTLV-1 EVs (2 k, 10 k, and 100 k; HUT102) were used to treat **a** CCF-STTG1, **b** MDM (THP-1 + PMA), **c** SHSY-5Y, and **d** MDM (U937 + PMA). Supernatants were collected 5 days after, enriched for EVs using nanotrap nanoparticles (NT80/82), and evaluated for presence of proinflammatory cytokines (IL-8 and IL-6) via Western blot analysis. HTLV-1 EVs (2 k, 10 k, and 100 k; HUT102) were exposed to none (untreated control), RNase A, or DNase I and then used to treat **e** CCF-STTG1 (Astrocytes; upper left panel) and mDCs (THP-1 cells + GM-CSF (50 ng/mL) + IL-4 (1000 U/mL) + TNF-α (20 ng/mL) + ionomycin (200 ng/mL); upper right panel). After 5 days of incubation, supernatants were collected and incubated with NT80/82 for EV enrichment. Western blots analysis was performed to determine protein expression of IL-8, RANTES, GAPDH, and Actin. Densitometry analysis of the RANTES bands for astrocytes (lower left panel) and mDCs (lower right panel) was carried out using ImageJ analysis software and subtracting the background of each membrane

In order to examine the potential role of HTLV-1 EVs in progression of neurodegeneration as observed in HAM/TSP patients, it is important to evaluate the BBB, composed of brain microvascular endothelial cells, pericytes, astrocytes, and together with the microglia/macrophage and neurons form the neurovascular unit. The hypothesis was whether HTLV-1 EVs were able to cross the BBB, then recipient cells in the CNS would respond to viral cargo (i.e., Tax and Env gp61/46) by secretion of cytokines involved in inflammation. We have recently shown that HTLV-1 EVs are capable of increasing proviral DNA in the brain of NOG mice and elicit effects on recipient cells (i.e. agglutination and viral transmission) [35]. In Fig. 5, individual cultures of cells typically involved in neuroinflammation such as astrocytes (CCF-STTG1), monocytic cell-derived macrophages (MDM; THP-1 and U937), and neurons (SHSY-5Y) were incubated with HTLV-1 EVs (2 k, 10 k, and 100 k). Western blot analysis was performed on the supernatant of each culture and probed for the presence of IL-8, IL-6, and RANTES. We observed an abundance of IL-8 from astrocytes treated with 2 k (lane 3), 100 k EVs (lane 5), and a less intense band but still present for 10 k EVs (lane 4); however, IL-6 was mostly absent (Fig. 5a). Data in Fig. 5b show that MDM cells ubiquitously expressed IL-8 (lanes 1–5), however the highest abundance was upon 2 k treatment (lane 3). Trace levels of IL-6 were observed in all conditions (lanes 1–4), except upon 100 k EV treatment (lane 5) and absence of RANTES across all treatments were observed (data not shown). In contrast, neurons did not express IL-8 or RANTES (data not shown), but they expressed the highest levels of background IL-6 in the controls (lanes 1 and 2). Neurons have been shown to express IL-6 under normal physiological conditions [89] and, therefore this higher background levels of IL-6 in controls is expected. Interestingly, the IL-6 levels decreased the most upon treatment with 2 k EVs compared to lane 2 (Fig. 5c). Finally, macrophages did not express either cytokine upon treatment with EVs (Fig. 5d). Overall, both astrocytes and MDM (THP-1) cells showed increased expression of IL-8, a cytokine shown to be involved in cell migration, angiogenesis, and metastasis in cancer cells [90–92]. Next, we performed Western blots to detect RANTES, IL-8, GAPDH, and ACTIN. In Fig. 5e, we observed that EVs elicited expression of RANTES in Astrocytes and monocytic cell-derived dendritic cells (mDCs). Addition of RNase A resulted in a slight decrease in the expression of RANTES. Addition of DNase I abolished expression of RANTES in Astrocytes and almost completely in mDCs. IL-8 expression was slightly increased upon addition of EVs treated with RNase A and DNase I in Astrocytes, but it was completely absent in all lanes for mDCs. GAPDH levels were affected for 2 k EVs in Astrocytes, and for 10 k > 2 k EVs for mDCs. These findings suggest that the EV cargo is sensitive to DNase I, potentially due to presence of nucleosome fragments that can serve as DAMPs. The depletion of EV-associated DNA or RNA has the potential to reduce inflammation.

### HTLV-1 viral transmission is enhanced by 100 k HTLV-1 EVs in monocytic cell-derived dendritic cells

**Fig. 6:**
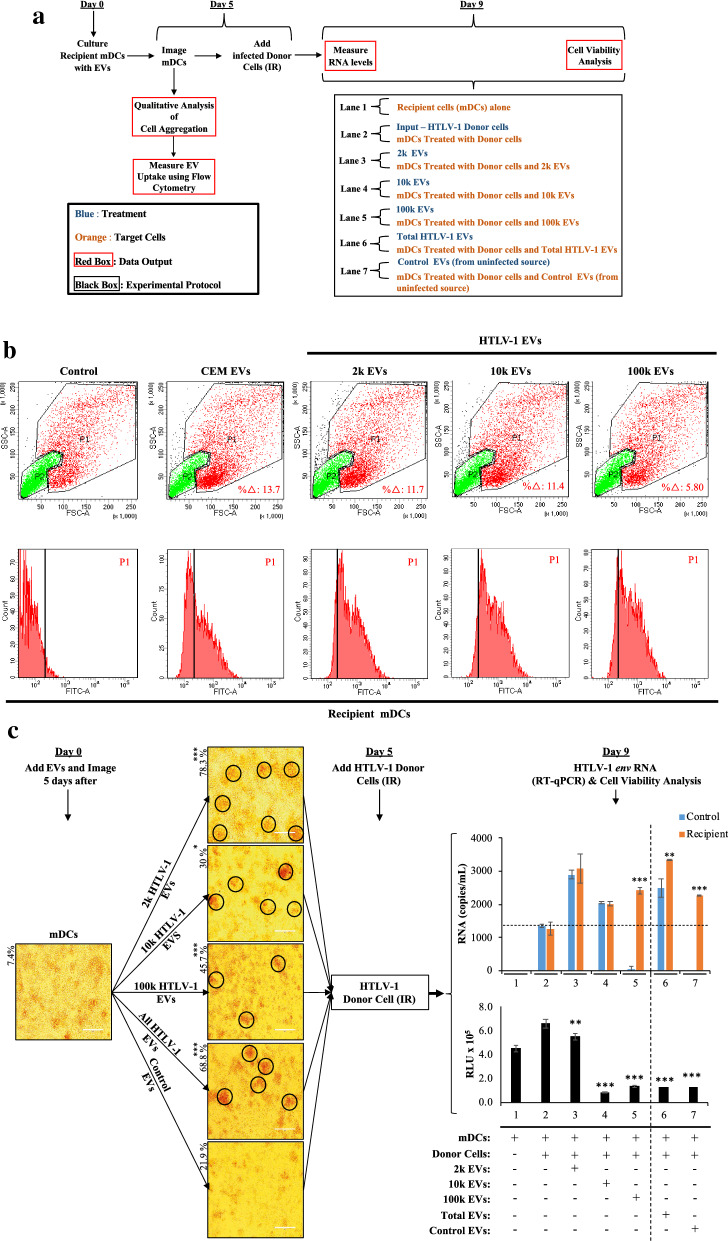
HTLV-1 100 k EVs enhance viral transmission in dendritic cells. **a** A diagram representing a timeline of the experimental design and treatment conditions from day 0 to day 9. **b** Flow cytometry analysis of the EV uptake by mDCs was performed in biological duplicate. Dot plots (upper panels) contain a P1 (red) and P2 (green) gated mDC populations, where the P1 was used to evaluate percent change (% ), increases in size and morphology, of P1 control compared to EV treatment. The control mDC populations in P1 and P2 are 33.7% and 49.9%, respectively. Histograms (lower panel) show increase in fluorescence intensity (right shift) as a function of EV uptake by mDCs. **c** Recipient mDCs were incubated for 5 days with various populations of HTLV-1 EVs (2 k, 10 k, and 100 k EVs) and CEM EVs (control EVs) at a ratio of 1 cell to 10,000 EVs. This was followed by microscopic analysis. Images are representative of three independent experiments. Following microscopy, recipient cells were then treated with irradiated HUT102 cells (HTLV-1 Donor Cells; 10 Gy) and fresh exo-free media for 4 additional days. RNA was isolated from recipient cell pellets and quantitated by RT-qPCR for viral *env* RNA. The RNA levels for the EV input are denoted by blue bars (control), while recipient cells treated with the input EVs by the orange bars (Recipient). The recipient mDC viability was analyzed at day 9 (cultured in biological triplicates). Vertical dashed bar (---) separates additional control EVs on the right-hand side for Total EVs (from HUT102) and Control EVs (from CEM). A two-tailed student t-test was used to compare control cells with recipient cells and to evaluate statistical significance with “*” for p-values ≤ 0.05, “**” for p-values ≤ 0.01, and “***” for p-values ≤ 0.001, indicating the level of significance

DCs have been shown to play an important role in viral transmission of HTLV-1 in mucosal tissues during breastfeeding and sexual intercourse [93]. We have shown that HTLV-1 infected cells secrete EVs containing viral proteins and RNA, with functional roles in modulation of cell-to-cell contact and viral transmission on uninfected cell lines, PBMCs, and in humanized infected NOG mice [35]. In Fig. 6, we evaluated the effects of EVs in monocytic cell (THP-1)-derived dendritic cells (mDCs) via use of flow cytometry, microscopy, RT-qPCR, and cell viability assays. The experimental design and timeline were described in detail in Fig. 6a. We performed FACS analysis on a 5-day old co-culture of mDCs with HUT102 EVs (2 k, 10 k, and 100 k EVs), CEM EVs, or no EVs (as control) to evaluate EV uptake by recipient cells (Fig. 6b). The live mDC populations were gated and denoted by “P1” (Control; Fig. 6b), which represented 33.7% of the total measured events (10,000 events total). The dead cell mDC population (P2), representing 49.9% of the total measured events, was identified and gated based on the low fluorescein isothiocyanate (FITC) and forward scatter (FSC) light intensity (data not shown); however, this is also observed in the side-scatter (SSC) *versus* FSC plots in Fig. 6b. The resulting Dot plots showed a 13.7% (CEM EVs), 11.7% (2 k EVs), 11.4% (10 k EVs) and 5.8% (100 k EVs) increase in the P1 mDC population, suggesting that EV treatment caused increases in granularity and size. The size increase is as also potentially due to increased cell aggregation, as observed previously. Additionally, we observed in histograms display an increase in fluorescence in the P1 population, as evidenced by the shift to right on the y-axis (FITC-A). This suggests that mDCs are uptaking or binding to all EVs types, with a slightly higher affinity for HTLV-1 EVs. In Fig. 6c (Day 5), we show that HTLV-1 EVs also caused increased cell agglutination in mDCs at day 5 post-treatment. Interestingly, we observed that treatment with 2 k HTLV-1 EVs yielded the highest level of cell clusters 78.3% (p-value ≤ 0.001) (Fig. 6c; Day 5, black circles), followed by 100 k EVs 45.7% (p-value ≤ 0.001), and the least with 10 k EVs 30% (p-value ≤ 0.05). Treatment with all populations of HTLV-1 EVs (All HTLV-1 EV; positive control) yielded an increase in agglutination (68.8%; p-value ≤ 0.001) as expected and treatment with EVs from uninfected CEM cells (All CEM EVs; negative control; 21.9%) had no significant increase. Here, virus was introduced by adding HTLV-1 infected cells (HUT102; donor cells) treated with ionizing radiation (IR; 10 Gy) to inhibit cellular replication in donor cells.

Nine days after initial treatment with EVs, RT-qPCR was performed to examine if particular EV populations also yielded changes in HTLV-1 *env* RNA levels on recipient mDCs (Fig. 6c; Day 9; upper panel). As expected, uninfected recipient mDCs had no HTLV-1 *env* RNA (lane 1) and donor cells alone (lane 2; Control) or with mDCs (lane 2; Recipient) resulted in about 1,360 *env* RNA copies/mL. The treatment with 2 k HTLV-1 EVs showed a twofold increase (3,085 copies/mL) in *env* RNA (lane 3; Recipient) compared to recipient cells with donor cells; however, 2 k HTLV-1 EVs alone (lane 3; Control) was not significantly different from RNA levels in recipient mDCs. The treatment with 10 k HTLV-1 also resulted in increased RNA levels (2,050 copies/mL; lane 4) compared to recipient cells with donor cells, with no significant difference between control and recipient mDCs. Interestingly, HTLV-1 *env* RNA levels in 100 k EVs where the lowest among all EV populations, with only 49 copies/mL (lane 5; Control). However, pretreatment with 100 k EVs resulted in a statistically significant increase of HTLV-1 *env* RNA by 2-logs to 2,411 copies/mL in the co-culture of mDCs with donor cells (lane 5; Recipient). The treatment with All HTLV-1 EVs also yielded higher levels of RNA in mDCs (3,347 copies/mL) than in control (2,496 copies/mL; lane 6). Finally, use of All CEM EVs caused a statistically significant increase in *env* RNA (lane 7; Recipient) similar to 100 k EV treatment. Overall, the data suggests that 100 k HTLV-1 EVs facilitate HTLV-1 Viral transmission in mDCs, despite observations of 2 k HTLV-1 EVs causing highest agglutination of mDCs. The 100 k HTLV-1 EVs are composed of less densely packed particles, of potentially smaller size, characteristics which may have facilitate uptake by mDCs and prime them for infection.

Interestingly, when cell viability was evaluated on three biological replicates, it was consistently observed that addition of EVs affected viability of mDCs (Fig. 6c, Day 9; lower panel). The 2 k HTLV-1 EV population caused the smallest decrease in cell viability (lane 3). Both 10 k and 100 k HTLV-1 EVs significantly decreased cell viability (lanes 4 and 5, respectively). We also observed that addition of All HTLV-1 EVs (Total EVs) and All CEM EVs (Control EVs) also caused a decrease in viability (lanes 6 and 7, respectively). A higher cell viability would result in an increased number of cells, which could explain the observed increased agglutination of mDCs upon 2 k treatment. The decrease in viability may suggests higher cellular stress, potentially resulting in cellular conditions that would promote increased viral transcription, such as in the case of 100 k HTLV-1 EVs (Fig. 6c; Day 9, lane 5). Collectively, these data indicate that all three populations HTLV-1 EVs enhance viral spread in mDCs, however, with increased efficiency upon pretreatment with the 100 k EV population.

### In vivo priming with select HTLV-1 EV populations increases susceptibility of specific tissues to HTLV-1 infection

HTLV-1 EVs had differential effects on uninfected recipient cells (i.e., mDCs). The 2 k EV populations caused more elevated numbers of cell agglutination compared to 10 k and 100 k. However, we observed increased susceptibility to infection on uninfected mDCs upon priming with 100 k HTLV-1 EVs. Other EV populations (i.e., 2 k and 10 k) also resulted in elevated HTLV-1 *env* RNA levels on newly infected cells, when compared to HUT102 donor cells. Since, all HTLV-1 EVs showed potential to cause increased cell-to-cell contact and increases of HTLV-1 *env* RNA, we examined the effects of each EV population on a humanized NOG mouse model, as described previously [35].

**Fig. 7:**
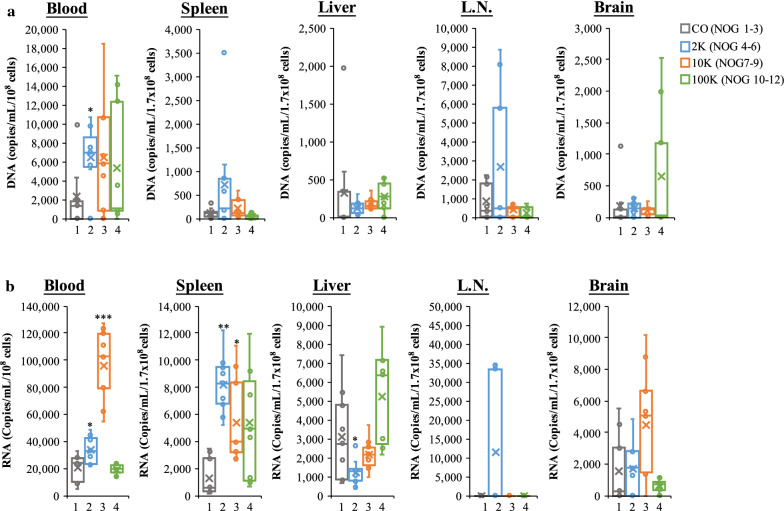
HTLV-1 2 k EVs promote enhanced viral spread. **a** Presence of proviral DNA (using qPCR for *env* region) from blood and tissues from multiple organs (Spleen, Liver, L.N., and Brain) of NOG mice (n = 12) treated with control (NOG 1–3), 2 k (NOG 4–6), 10 k (NOG 7–9), and 100 k (NOG 10–12) EVs followed by HUT102 donor cells (IRed) treatment 5 days later. **b** Presence of RNA (using RT-qPCR *env* RNA) from blood and tissues of NOG mice (n = 12) treated with control (NOG 1–3), 2 k (NOG 4–6), 10 k (NOG 7–9), and 100 k (NOG 10–12) EVs followed with HUT102 donor cells (IRed) treatment 5 days later. A two-tailed student t-test was used to evaluate statistical significance with “*” for p-values ≤ 0.05, “**” for p-values ≤ 0.01, and “***” for p-values ≤ 0.001, indicating the level of significance compared to control

Twelve humanized NOG (hu-NOG) mice were initially treated with 2 k, 10 k, or 100 k HTLV-1 EVs (10 μg in 250 L of PBS) and 5 days later treated with HUT102 donor cells (10 Gy; IRed). Peripheral blood and tissues ((i.e., Liver, Spleen, Lymph Node (L.N.), and Brain)) were collected three weeks later and processed for qPCR analysis of proviral DNA and RT-qPCR analysis of *env* RNA in each tissue. In Fig. 7a, we show that HTLV-1 proviral DNA in the Blood of NOG mice was increased by 2 k, 10 k, and 100 k HTLV-1 EVs. However, 2 k EVs caused the most consistent increase, with statistical significance (t-test: p-value ≤ 0.05). The Spleen showed a potential increase in proviral DNA when animals were pre-treated with 2 k EVs, but not with 10 k or 100 k EVs. The Liver seemed less susceptible to pretreatment with EVs and HTLV-1 infection, as no increase over control was observed, for 2 k (NOG 4–6), 10 k (NOG 7–9), and 100 k (NOG 10–12) EVs. HTLV-1 proviral DNA in the L.N. was increased with 2 k EVs only and, finally, proviral DNA levels in the Brain were increased above control when treated with 100 k EVs. The overall trend in viral DNA spread in vivo was 2 k > 10 k > 100 k. However, tissues such as the Brain showed potential for 100 k EVs to elicit increased viral transmission (100 k > 2 k > 10 k).

We next further examined the tissues of the twelve humanized NOG mice by evaluating the levels of viral RNA transcription in vivo to determine if a particular EV population resulted in productive or non-productive infection. In Fig. 7b, we observed that the Blood of NOG mice treated with EVs had a significant average increase of 1.7 and 4.6-fold higher *env* RNA levels with 2 k and 10 k, respectively. No changes to *env* RNA were observed in mice treated with 100 k EVs. An eightfold and fivefold average increase of *env* RNA in the Spleen was observed for 2 k and 10 k EVs, respectively. No significant change was observed with 100 k EV pretreatment in the Spleen. The Liver and L.N. was not noticeably affected by EVs, except for a decrease in *env* RNA in the liver and increase in the L.N. with 2 k EV pretreatment. Finally, the Brain was not affected by EV pretreatment, except for a 2.9-fold increase in 10 k EV treated mice. Overall, the 10 k HTLV-1 EV population showed increased potential to mediate productive viral transmission in the Blood, and 2 k EVs in the Spleen and potentially L.N.

## Discussion

EVs represent a potential mechanism of autocrine, paracrine, and endocrine cell-to-cell communication that pathogens may exploit to mediate disease [35, 38, 41, 57, 66, 94]. Viruses, such as HTLV-1, rely heavily on host–pathogen interactions for their viral life cycle. HTLV-1 is particularly reliant in the hijacking of cellular machinery for viral replication and the perpetuation of its genome via spread from cell-to-cell. Transmission of the HTLV-1 virion occurs with higher efficiency upon the direct contact between immune cells [95], however, our recent data suggests that EVs may play a pivotal role in 1) enhancing the efficiency of viral spread by promoting cell-to-cell contact [35]; and 2) facilitating viral spread to tissues across the human body and in animal model [35, 41]. Here we also found that distinct EV populations secreted from HTLV-1 infected cells (HTLV-1 EVs) may potentiate inflammatory and cellular damage associated in recipient cells.

We suspected that HTLV-1 may preferentially utilize EV populations derived from autophagy related pathways and characterized by a larger vesicle size or, alternatively, by more densely packaged cargo (i.e., 2 k or 10 k EVs). EV biogenesis from cellular pathways such as autophagy may generate vesicles with the aforementioned characteristics due to their roles in degradation of large amounts of intracellular waste, requiring larger vesicles to encapsulate the debris [38, 72]. It is known that HTLV-1 can modify autophagy by inhibiting the fusion of degradative enzymes in the lysosome with the waste cargo in the autophagosome [50, 96]. This inhibition would prevent degradation of intracellular waste (i.e., accumulating viral proteins, cytokines, and other products), captured in autophagosomes, and secretion into the extracellular domain. Our data suggests that indeed the virus utilizes EVs of different density/sizes (i.e., 2 k, 10 k, and 100 k) to package viral proteins (Fig. 1). We hypothesized that the 2 k EV population (i.e., either larger or more densely packed with cargo) is generated predominantly by the autophagy pathway. The 10 k EV population may also be from the same pathway, and we suspect that autophagy may contribute in part (20%) to the generation of the 10 K vesicles, while 80% may be derived from vesicle exocytosis, such as in the case of Microvesicles [60, 97, 98]. Further purifications of these EVs in the future could clarify this discrepancy. Finally, the 100 k EVs are smaller in size and of the lowest density out of the three populations, and we suspect it is derived via pathways, such as ESCRT-dependent pathways, that generate smaller vesicles (i.e., exosomes) with specific cargo captured by ubiquitination [66]. However, the presence of viral proteins in either of these EV fractions may be indicative of viral hijacking cellular pathways that may modify cargo and function of each EV population. EVs are ubiquitously expressed by cells and understanding how viruses modulate these vesicles, highly capable of cell-to-cell communication, may be intrinsic to further understanding pathogenesis.

HTLV-1 seropositivity in clinic may be confirmed by presence of the viral protein Gag (p19) [99–101]. However, HTLV-1 Tax is highly immunogenic and has been detected in brain and spinal cord from HAM/TSP biopsies [102–105]. Therefore, it was important to further investigate presence of other viral antigens (i.e., p19 and Tax) to understand the extent at which EVs are involved in HTLV-1 immune deregulation. HTLV-1 EVs, from the CSF of HAM/TSP patients, have already been shown sensitize targets cells for Tax-specific Cytotoxic T lymphocytes (CTLs) lysis [41].

HTLV-1 EVs may modulate immunity by promoting release of inflammatory cytokines and chemokines. HTLV-1 proteins may also elicit antiviral responses via recognition of pathogen associated molecular patterns (PAMPs) [106–108]. Moreover, HTLV-1 has a strong oncogenic potential driven primarily by Tax [7]. We previously detected Tax in EVs [35, 54] and, as indicated above, observed the potential for EV-associated Tax to activate the immune system [41]. Here we detected presence of Tax in the 2 k and 10 k EV subsets (Fig. 1). Overall, the data suggests that HTLV-1 infected cells and individuals may experience an EV-mediated dynamic interplay between activation of innate immune antiviral responses (via 10 k and 100 k EVs) and Tax-mediated damage (via 2 k and 10 k EVs). It is important to note that we originally used mass spectrometry to evaluate protein content in HTLV-1 EVs and detected Tax only in 2 k EVs. However, we performed multiple western blots from biological replicates which suggested presence of Tax in both 2 k and 10 k EVs, and less frequently in 100 k EVs. This is not surprising since the expression of Tax in ATLL patients has been characterized as a transient expression [109] and dynamically tied to gene regulation by Tax (positive feedback loop) and HBZ (negative feedback loop) [110–113]. It is possible that the samples used for our proteomic analysis were from HTLV-1 infected cells expressing low-levels of Tax and therefore packaged into vesicles generated by autophagy (i.e., in autophagosomes). Some of our recently published data using IR to activate HTLV-1 transcription suggests that this activation yields an increase in overall EV secretion and Tax packaging into a wider array of EVs [35]. We can use this method of activation to modulate Tax expression in infected cells and packaging into distinct EV populations. Therefore, our future studies will use IR as a tool to modulate Tax expression and quantitative proteomics to evaluate the dynamic expression of Tax and other viral proteins in various EVs.

Proteomic analysis of HTLV-1 EVs also showed presence of several cellular proteins in 2 k HTLV-1 EVs that support the hypothesis that the 2 k EVs are derived from autophagy. For instance, some of the major pathways uniquely detected in the 2 k EVs were associated with phagosome formation (i.e., autophagy and autophagosome formation), cell cycle regulation, ribosome biogenesis, Ras GTPase binding, and Parkinson’s disease (Fig. 2). Background low levels of apoptosis could also be responsible for the presence of several cellular proteins in 2 k EVs. However, we expect that these apoptotic vesicles are not derived from dying cells [35, 56]. These findings revealed the possibility that HTLV-1 EVs may also carry important cytokines and chemokines with the potential to further regulate the immune system of an infected host. Indeed, HTLV-1 EVs carried unique levels of distinct cytokines encapsulated and bound to the surface of EVs. The potential importance of cargo being encapsulated or membrane-bound may be that vesicular membrane would be able to protect encapsulated cargo from degradation and potentially enter targets cells more easily by vesicular fusion with target cells. In contrast, membrane-bound cargo may be able to interact with the EV environment more easily and serve as localization signals, however these are also more susceptible to degradation. Both 2 k and 10 k EVs contained cytokines involved in inflammatory responses that could lead to cellular damage by persistent macrophage activation (i.e., 2 k EVs; MIP-1a encapsulated) and pro-inflammatory signaling (10 k EVs; IL-18 encapsulated) (Fig. 2). The 100 k EVs contained high levels of a chemokine involved in recruitment of myeloid cells (RANTES; encapsulated) (Fig. 2), suggesting the potential to activate the immune system, however not necessarily initiate responses that would lead to tissue damage. Interestingly, all three HTLV-1 EV populations contained high levels of IL-33 bound to the membrane surface (Fig. 2). IL-33 is a ubiquitously expressed cytokine important in regulating the immune system in protective processes such as tissue repair [79]. However, the presence IL-33 in EVs from HTLV-1 infected cells may serve these vesicles as a biomarker to evade the detection of the immune system and engulfment by, for instance, activated macrophages. This is in line with HTLV-1 evolution to use intrinsic cytokines and pathways to bypass the immune system and elicit recruitment of cells to enhance viral spread and collateral damage in cases of chronic immune activation. It is also possible that 2 k and 10 k EVs have evolved to become part of the viral life cycle by inducing increased damage, recruitment of uninfected immune cells, and potential viral spread. The 100 k EVs population may be the milder less dense version of the three and, therefore, it may harbor the possibility of serving as a source of vaccine by initiating a mild immune response that can be controlled. This is evident by presence of only some of the key HTLV-1 immunogens inside or on the surface of these EVs (Fig. 2). Our future research aims to further investigate the potential of the 100 k EVs as a source of vaccine and whether these EVs are sufficient as stand-alone or if they may be used as an adjuvant in vaccines.

We previously observed that EVs from HTLV-1 infected cells induced enhanced cell-to-cell contact. This was evaluated in multiple biological replicates and via quantitation of the total number of cells in culture (n ≥ 500, per well), as well as the total number of cells aggregated together. After 24 h in culture, we observed that 59% of the uninfected cells (treated with fluorescently labeled HTLV-1 EVs) were aggregated together [35]. Additionally, EVs isolated from HAM/TSP patient PBMCs (which were Tax positive) induced Tax-specific CTL lysis at levels similar to purified immunogen [41]. These data pointed towards the existence of important functional roles of EVs in HTLV-1 pathogenesis related to its viral spread and CNS inflammation (especially since EVs containing Tax were also found in HAM/TSP patient CSF). Therefore, it was important to understand if an EV population was responsible for these functional effects. All three EV populations (i.e., 2 k, 10 k, and 100 k) were able to differentially enhance cell-to-cell contact in uninfected recipient T-cells (Fig. 3) and mDCs (Fig. 6). When evaluating EV uptake, it was not surprising that that all EVs types (infected and uninfected) were uptaken by mDCs because of their specialized phagocytic functions. However, it was interesting that despite the overall uptake of EVs, only HTLV-1 EVs elicited functional effects. These data validate our ongoing observations about the relevance of HTLV-1 EVs (and cargo) to disease progression. In addition, it is possible that distinct EV populations have target cell specificity, where 2 k/10 k EVs enhanced effects on T-cells and 100 k EVs on mDCs. It is possible that EV target affinity may be directly related to membrane-bound cargo directing a fate towards the peripheral blood (and associated tissues) or the CNS. Yet, cargo within EVs may ultimately dictate secondary functional effects, such as immune activation, tissue repair, or damage.

Interestingly, EVs were also able to elicit functional effects related to tissue damage on a model of the BBB using mesenchymal stem cells and tubular formation by endothelial cells. Tubule deterioration was most significant upon treatment with 2 k and 10 k EVs, evident mainly after extended time periods (Fig. 4). This time-dependent HTLV-1 EV-induced damage suggests presence of cargo that not only induces direct damage (i.e., PAMPs and DAMPs), but may also induce secondary damage by the expression of cellular genes that activate cell death pathways. This secondary damage would require time for signal transduction, gene expression, and positive feedback loops to potentiate damage. Cell death related signaling has already been linked to EVs. More specifically, DNA fragments bound to histones (i.e., nucleosomes) have been found associated with EVs, with the potential to induce strong DAMP associated signaling [83, 87]. We found that 2 k and 10 k EVs contained all histones (i.e., H1, H2A, H2B, H3, and H4), therefore have potential to act as DAMPs (Fig. 4). HTLV-1 EVs may carry DAMPs differentially in subpopulations since our data suggests that 2 k EVs carry membrane-bound DNA fragments with all histones; whereas 10 k EVs carry encapsulated DNA/histones, and 100 k EVs encapsulated DNA fragments (Fig. 4). Additionally, the anti-parrel correlation between EV-dose and DNA levels suggests at higher EV concentrations, the proteinase k used may be insufficient to digest histones and expose DNA for detection by PCR. Altogether, these data validate our observations of 2 k and 10 k EV effect on tubule deterioration and that encapsulated DAMPs (i.e., histones) in 10 k EVs may potentiate damage via secondary effects.

As discussed earlier, cytokines and DAMPs found associated with HTLV-1 EVs (2 k and 10 k) have known roles in immunomodulation. Cytokines such as MIP-1a (in 2 k EVs), IL-18 (in 10 k EVs), and RANTES (in 100 k EVs) have been shown to have roles in damage and CNS inflammation (i.e., recruitment of immune cells and activation of macrophages) that could affect the BBB [114–116]. More specifically, MIP-1a, IL-18, and RANTES have been associated with viral infection, Alzheimer’s disease, and traumatic brain injury, respectively. However, presence of MIP-1a in the neurovascular junction may lead to secondary damage related to activation of macrophages and subsequent TNF-α release. Therefore, the HTLV-1 2 k EV has strong potential to activate the immune system, and in combination with DAMPs (i.e., histones) cause increase damage to the BBB. On the other hand, RANTES promotes migration of peripheral leukocytes, by binding to the CCL5 receptor, to the BBB resulting in increased permeability [115]. Along these lines, we have observed that both 2 k and 100 k EVs permeabilized the integrity of the BBB (data not shown) and had effects on recipient cells in the neurovascular unit (Fig. 5). It may be possible that 2 k and 100 k HTLV-1 EVs target astrocytes during acute exposure as evidenced by increased secretion of the IL-8 proinflammatory cytokine. However, future studies will aim at further examining the effects of EVs on the BBB and neurovascular unit during chronic EV exposure, especially since symptomatic HTLV-1-infected patients (i.e., HAM/TSP patients) live with the virus for prolonged periods prior to development of HAM/TSP.

Finally, use of the hu-NOG model allowed validation of our in vitro observations regarding the potential for 1) 2 k/10 k EVs to be the most pathogenic (2 k > 10 k); and 2) 100 k EVs the most immunogenic and potentially permeable through the BBB. Indeed, we detected an overall higher level of proviral DNA in the Blood, spleen, and L.N. of hu-NOG mice post-treatment with 2 k EVs (Fig. 7). In contrast, 100 k EVs increase proviral DNA in the Brain. It is important to note that viral RNA levels in the mice may be used as a marker of productive viral infection, and only 2 k and 10 k EVs enhanced viral RNA levels (i.e., Blood, Spleen, and L.N.). The lack of a significant increase in viral RNA levels in the brain post-acute treatment with 100 k EVs suggests that these EVs may promote increased viral entry to the CNS, however this results in a nonproductive infection. Damage and efficient viral spread to the brain by HTLV-1 may require chronic CNS exposure to EVs or alternatively, the use of total EVs (2 k, 10 k, and 100 k EVs) [35].

## Conclusions

Collectively, these findings suggest that HTLV-1 pathogenesis may depend on EVs for enhanced and efficient viral spread. The 2 k EVs, and to a lesser extent 10 k, could be considered the “bad” EVs which cause damage. In contrast, the 100 k EVs has the potential to do less damage, which may be mitigated. Our future experiments are evaluating the use of 100 k HTLV-1 EVs as a source of vaccine that would prime the immune system, but not necessarily induce damage. The potential use of EVs in therapeutics has previously been explored in the solid tumor biology as possible vaccines, or as an adjuvant, which could potentially also be used effectively in liquid cancer.

## Materials and methods

### Cells

The HTLV-1 infected cell (HUT102), uninfected T-cell (CEM and Jurkat), promonocytic cells (THP-1 and U937), and astrocyte cell lines (CCF-STTG1; ATTC CRL-1718™) were grown for 5 days in RPMI-1640 media (Quality Biological). RPMI Media was supplemented with 10% heat-inactivated exosome-free fetal bovine serum (FBS) (Peak Serum), 2 mM l-glutamine (Fisher Scientific), 100 U/mL penicillin (Quality Biological), and 100 μg/mL streptomycin (Quality Biological) as previously described [35]. On day 0 and 1, THP-1 cells were supplemented for with GM-CSF (50 ng/mL), IL-4 (1000 U/mL), TNF-α (20 ng/mL) + ionomycin (200 ng/mL) to differentiate into monocytic cell-derived dendritic cells (mDCs). To obtain monocytic cell-derived macrophages (MDM), THP-1 or U937 cells were treated with 1 μL/mL of PMA (working stock at 100 μM; on day 0 and 1). Neurons (SHSY-5Y; ATCC CRL-2266™) were cultured for 5 days in 50% EMEM media (ATCC 30–2003™) supplemented with 10% FBS. Bone marrow derived Mesenchymal Stem Cells (MSCs) were cultured in Mesenchymal Stem Cell Basal Medium (ATCC PCS-500–030™) supplemented with Mesenchymal Stem Cell Growth Kit for Bone Marrow-derived MSCs (ATCC PCS-500-041™). Cell counts were performed using KOVA® Glasstic Slide with Counting Grids hemocytometer (KOVA® Cat# 87,144) with trypan blue (1:1) for a total count of 1 × 10^6^ cells/mL for Western blots and 5 × 10^5^ cells/mL for cell viability assays. Cell cultures were incubated at least for 5 days at 37 °C with 5% CO_2_ prior to isolation of EVs, as described below.

### Reagents and antibodies

Primary antibodies against proteins of interest used in Western blot assays were: α-Tax (Tax antibodies 169, 170, and 171 monoclonal mouse (generous gift of Dr. Scott Gitlin, University of Michigan) [35, 54]; α-p19 (Santa Cruz Biotechnology, sc-1665); α-gp61/46 (NIH AIDS Reagent Program Cat. # 1578); LC3-1/LC3-II (Santa Cruz Biotechnology, Cat. # SC-398822); p62 autophagy marker (Cell Signaling Inc., Cat. # 5114S); α-CD45 (Santa Cruz Biotechnology, Cat. # SC-1123); α-CD43 (Santa Cruz Biotechnology, Cat. # SC-70682); α-ICAM-1 (Santa Cruz Biotechnology, Cat. # SC-8439); α-LFA-1 (Santa Cruz Biotechnology, Cat. # SC-374172); α-CD63 (Santa Cruz Biotechnology, Cat. # SC-365604); α-CD63 tetraspanin marker (SBI System Biosciences, Cat. # EXOAB-CD63A-1); α-histone H2B (Cell Signaling Technology, Cat. # 12,364); α-histone H2A (Santa Cruz Biotechnology, Inc., Cat. # SC-10807 and abcam, Cat. # ab18255); α-histone H3 (Santa Cruz Biotechnology, Inc., Cat. # SC-10809); α-histone H4 (Cell Signaling Technology, Cat. # 2592); α-histone H1 (GeneTex, Cat. # GTX114462); IL-8 (Santa Cruz Biotechnology, Cat. # SC-376750); IL-6 (Santa Cruz Biotechnology, Cat. # SC-28343); RANTES (Santa Cruz Biotechnology, Cat. # SC-514019); GAPDH (Santa Cruz Biotechnology, Cat. # SC-48166); and α-Actin (Abcam; ab469900).

### X-ray irradiation

Ionizing radiation (IR) based treatments were performed using an X-Ray Irradiator (RS-2000 X-Ray Irradiator; Rad Source, Suwanee, GA, United States) for a total of 10 Gy per sample. Irradiated HUT102 cells were allowed to incubate for 5 days in order to isolate IR treated EVs for cell viability studies. IR was also used to treat HUT102 cells to induce cell cycle arrest (Donor cells). The Donor cells were then used as a source of HTLV-1 for the evaluation of viral transmission in vitro and in vivo*.*

### EV isolation via differential ultracentrifugation

HUT102, ATL-16, and CEM cell cultures were grown in 1-L roller bottles (Corning Inc.), with 100 mL of initial cellular material, to which approximately 100 mL of complete RPMI media added daily up to 500 mL. The cells were allowed to grow for 5 additional days at 37 °C with 5% CO_2_. Cells were then separated from supernatant by low-speed centrifugation (300–400×*g* for 10 min) and supernatants were transferred into eight ultracentrifugation tubes (each with 22.5 mL of supernatant). The tubes were placed in a Ti70 (Beckman) rotor and spun at 2000×*g* (2 k) for 45 min. The resulting supernatant was added to a new set of eight ultracentrifugation tubes (each with 22.5 mL of supernatant) and weighed for further ultracentrifugation, while the pellets were resuspended with PBS (150 to 200 μL per ultracentrifuge tube) and consolidated into a single 5 mL tube yielding 2 k EVs. Next, the supernatant was spun at 10,000×*g* (10 k) for 45 min. The resulting supernatant was added to a new set of eight ultracentrifugation tubes (each with 22.5 mL of supernatant) and weighed for further ultracentrifugation, while the pellet resuspended with PBS (as specified above) and consolidated into a single 5 mL tube yielding the 10 k EVs. The final supernatant was spun at 100,000×*g* (100 k) for 90 min. The resulting supernatant was discarded, while the pellet was isolated with a PBS as described above and consolidated into a single 5 mL tube yielding the 100 k EVs. All isolated EVs were placed in a − 20 °C freezer for later downstream assays.

### EV enrichment using nanotrap particles (NTs)

Nanotrap particles (Ceres Nanosciences, Inc.) were used to enrich EVs from low volume, cell-free supernatant samples (1 mL) as previously described [35]. Briefly, a 30% slurry of NT082 (Ceres #CN2010), NT080 (Ceres #CN1030), and 1 × Phosphate Buffered Saline (PBS) were combined, and 30 μL was added to 1 mL of each sample supernatant. The samples were then rotated overnight at 4 °C to capture EVs. The resulting pellet was washed once with PBS and used for downstream experiments.

### ZetaView nanoparticle tracking analysis (NTA)

NTA was performed using ZetaView® Z-NTA (Particle Metrix) with its corresponding software (ZetaView 8.04.02). Calibration was done using 100 nm polystyrene nanostandard particles (Applied Microspheres) prior to sample readings at a sensitivity of 65 and a minimum brightness of 20. Automated quality control measurements including cell quality check and instrument alignment were performed prior to sample measurements. Instrument parameters were set as previously described [35, 42, 66] for each reading. For each sample, 1 mL of sample diluted in PBS was loaded into the cell. The size and concentration of particles were measured at 11 unique positions throughout the cell in three independent reads. After automated analysis and removal of any outliers, the mode diameter size and the concentration of the sample were calculated by the machine software. Data for NTA analysis was compiled with the corresponding manufacturer software, ZetaView 8.04.02, and plotted using Microsoft Excel 2019. The mode, defined as the size of the most abundant particles, was used as the measurement for size in our analysis.

### Fluorescent microscopy and EV labeling

EVs were isolated from 5-day-old HUT102 cell culture and separated into 2 k, 10 k, and 100 k EV populations. The fluorescent label of BODIPY™ 493/503 (Cat. # D3922; Invitrogen™) was mixed (1.5 μL) with EVs (50 μL) and allowed to incubate for 30 min at 37 ºC. A Pharmacia G-50 spin column (1 mL bed volume in PBS buffer; 2000 rpm/2 min; Sorval RT6000D) was used to filter out any unbound BODIPY, yielding 30 μL of labeled EVs. Resulting BODIPY EVs were quantified again by NTA (ZetaView) since concertation changes were expected due to labeling with BODIPY. The resulting EV concentrations were 6.20 × 10^10^ EVs/mL for 2 k, 2.03 × 10^11^ EVs/mL for 10 k, and 8.20 × 10^10^ EVs/mL for 100 k EVs. EVs were then added onto recipient cells normalized to volume (5 μL of each EV in 95 μL of cell culture at 5 × 10^5^ cells/mL) in biological triplicate. Treated cells were analyzed with an EVOS-FL-Auto microscope (Life Technologies).

### Transfection with siRNA

The Lipofectamine RNAiMAX reagent protocol (Life Technologies, Inc.) was used to transfect HUT102 cells with siRNA against CD45 and ICAM-1. Briefly, ∼0.5 × 10^6^ were seeded on a 24-well plate. The next day, Lipofectamine RNAiMAX reagent was diluted in Opti-MEM medium by adding 25 μL of Opti-MEM medium to 1.5 μL of Lipofectamine RNAiMAX reagent. siRNA was then diluted in Opti-MEM medium with 25 μL of Opti-MEM medium, and 1.5 μL of siRNA (10 μM) was used. Twenty-five microliters of the diluted siRNA were then added to 25 μL of the diluted Lipofectamine RNAiMAX reagent and allowed to incubate at room temperature for 5 min. Cell medium was then removed, and 50 μL of siRNA–lipid complex and ∼250 μL of fresh medium were added to the cells (the final concentration of siRNA used was 20 nm) and allowed to incubate overnight. The siRNA–lipid complex was then washed and removed, 0.25 mL of complete medium was added, and cells were incubated at 37 °C for 5 days. EVs from supernatants were isolated, labeled with BODIPY™ 493/503 (Cat. # D3922; Invitrogen™) and cleaned over G-50 spin column. The EVs were added at 1:20,000 ratio (Cell: EV ratio; over 2 days) and images were taken at days 3 using an EVOS-FL-Auto microscope (Life Technologies), under 40 × magnification with phase objective and fluorescence. The images were quantitated in technical and biological triplicates counting total cells in the field of view. To quantitate EV-mediated cell-to-cell contact, clumps of cells colocalized with EVs (denoted by fluorescence) were counted, as well as the total number of cells within each clump. The percent of total clumped cells was calculated against total cells (n > 500 for each condition).

### Cell viability

Recipient cells (5 × 10^5^ cells/mL) were cultured in technical triplicates in 100 μL of fresh EV-depleted RPMI media in a 96-well assay plate (Corning Inc.; Cat#: 3610) and treated with HTLV-1 and CEM EVs as specified for each experiment. Cells were allowed to incubate for 5 days and CellTiter-Glo reagent (Promega; Cat#: G7572) was used at a 1:1 ratio to detect cell viability on a GloMax Explorer (Promega). EV-depleted RPMI media alone was cultured and used for background measurements.

### Western blot analysis

CEM and HTLV-1 cells were harvested, pelleted, washed once in PBS and resuspended in lysis buffer containing 50 mM TrisHCl (pH 7.5), 120 mM NaCl, 5 mM EDTA, 0.5% Non-idet P-40, 50 mM NaF, 0.2 mM Na3VO4, 1 mM DTT, and 1 complete protease inhibitor mixture tablet/50 mL (Roche Applied Science). Lysate suspensions were incubated on ice for 20 min and vortexed every 5 min, followed by centrifugation at 10,000 × g for 10 min at 4 °C to pellet the cellular debris. Protein concentrations from the lysate supernatant were determined using Bradford protein assay (BIO-RAD). For Western blot analysis, cell lysates (15 µg); CEM EVs and HTLV-1 EVs (1 × 10^9^ EVs) were resuspended in 15 µL Laemmli buffer, heated 3 times for 3 min at 95 °C, and loaded onto a 4–20% Tris–glycine SDS gel. Subsequently, proteins were transferred onto PVDF membranes (Millipore) overnight at 50 mA. Membranes were then blocked for 30 min at 4 °C in blocking buffer containing 5% dried skimmed milk in PBS containing 0.1% Tween 20. Membranes were incubated overnight at 4 °C with primary antibody against specified proteins. The following day, membranes were washed and incubated with the appropriate HRP-conjugated secondary antibody for 2 h at 4 °C and developed with Clarity or Clarity Max Western ECL reagent (BIO-RAD, cat#1,705,061, cat#1,705,062, respectively). Luminescence was visualized on a ChemiDoc Touch Imaging System (BIO-RAD). Densitometry analysis of the bands was carried out using ImageJ analysis software, by subtracting the background of each membrane.

To analyze the histones associated with EVs, the HUT102 EV samples were isolated via DUC (2 k, 10 k, and 100 k) resulting in concentrations of 1.22 × 10^11^ particles/mL for 2 k, 4.30 × 10^11^ particles/mL for 10 k, and 2.63 × 10^10^ particles/mL for 100 k EVs. Next, 10 μL of each sample was heated to approximately 90 °C for 3 min and mixed with 8 μL of Laemmli buffer. Next, 18 μL of the EV/Laemmli buffer mixture were loaded onto a 4–20% SDS/PAGE and run at 100 mV for 15 min, followed by 150 mV for 40 min. The proteins were then transferred to PVDF membranes (Millipore) overnight. The transferred membranes were then blocked with 1.25% Bovine Serum Albumin Fraction V (dissolved in 1 × PBS containing 0.1% Tween 20) for 30 min. Subsequently, the primary antibodies for Histones (H1, H2A, H2B, H3, and H4) and Actin were diluted in 5 mL of PBS (containing 0.1% Tween 20 and 250 μL of 1.25% Bovine Serum Albumin Fraction V) at ratios as described by the manufacturer (see Reagents and Antibodies section) and allowed to incubate with the blots overnight.

### RNase A and DNase I EV treatment

Various HTLV-1 EV subpopulations (2 k, 10 k, and 100 k HUT102 EVs; 1:10,000 cells) were subjected to RNase A (Abcam) or DNase I (Promega, M6101) digestion for 1 h at 37 °C to remove any DNA or RNA outside of EVs. The RNase A and DNase I enzyme activity in the EV samples were removed by addition of TF and cleaned over G-50 spin column. These EVs and untreated EVs (control) were used to treat astrocytes (CCF-STTG1) and mDCs (THP-1 cells + GM-CSF (50 ng/mL) + IL-4 (1000 U/mL) + TNF-α (20 ng/mL) + ionomycin (200 ng/mL)). Cells (5 × 10^5^ cells/mL) were plated and treated with EVs at a ratio of 1:10,000. Supernatants were collected 5 days later and enriched for EVs using nanotrap nanoparticles (NT80/82). Western blot analysis was performed to assess the presence of proinflammatory cytokines (IL-8 and RANTES), GAPDH, and Actin.

### RNA Isolation and real-time qPCR

HTLV-1 RNA analysis was performed using total RNA isolated from either cell pellets or Nanotraped pellets. TRIzol Reagent (Invitrogen) was used according to manufacturer’s protocol. Total RNA was quantitated by a NanoDrop 1000 Spectrophotometer (Thermo Scientific) and used to generate cDNA with the GoScript Reverse Transcription System (Promega) using Oligo (dT). RT-qPCR analysis was done using 2 μL aliquots of undiluted cDNA, SYBR Green master mix (Bio-Rad), and specific primer sets were used: HTLV-1 env (env-Reverse 5′-CCA TCG TTA GCG CTT CCA GCC CC-3′, Tm = 64.4 °C; env-Forward 5′-CGG GAT CCT AGC GTG GGA ACA GGT-3′, Tm = 64.5 °C). Serial dilutions of DNA from HUT102 cells were used as quantitative standards. PCR conditions were as follows: for env primers 50 °C for 2 min, 95 °C for 2.5 min, then 40 cycles of: 95 °C for 15 s, 64 °C for 40 s. The quantification of samples was determined based on cycle threshold (Ct) values relative to the standard curve for each plate. Reactions were carried out in triplicate using the Bio-Rad CFX96 Real-Time.

### DNA isolation and RT-qPCR

DNA was isolated from 2 k (6.27 × 10^11^ particles/mL), 10 k (6.37 × 10^11^ particles/mL), and 100 k (8.47 × 10^11^ particles/mL) HUT102 EVs and HUT102 cell lysates (controls and standards) using the GoScript Reverse Transcription System (Promega) and concentration determined using NanoDrop 1000 Spectrophotometer (Thermo Scientific). EVs were evaluated in titration at volumes of 0.5, 1, and 2 μL for each EV. To evaluate if DNA bound to histones was present in the membrane of EVs or encapsulated, EV samples were treated with Proteinase K (Sigma, Cat# P4850) alone at 95 °C for 3 min, or with DNase I (Promega, Cat# M6101) and RNase A (Abcam, Cat# G117) at 37 °C for 1 h prior to addition of DNase Stop Solution (Promega, Cat# M199A) 65 °C for 5 min. DNA was isolated using the Wizard® Genomic DNA Purification Kit according to the manufacturer’s protocol (Promega Corporation) and quantified via qPCR using primers (IDT Technologies) specific for *GAPDH* (*GAPDH-*Forward 5′-TGT AGT TGA GGT CAA TGA AGG G-3′, Tm = 64.5 °C; and *GAPDH* Reverse 5′-ACA TCG CTC AGA CAC CAT G-3′, Tm = 64.5 °C). Serial dilutions of DNA from HUT102 cells were used as quantitative standards. PCR conditions were as follows: for *GAPDH* primers 50 °C for 2 min, 95 °C for 2.5 min, then 39 cycles of: 95 °C for 15 s, 50 °C for 30 s, and 72 °C for 40 s. The quantification of samples and reactions were carried out as described above.

### Mass spectrometry analysis

Eluates obtained from 2 k, 10 k, and 100 k HUT102 supernatants were first reduced by 200 mM dithiothreitol at room temperature for 15 min and then alkylated with 50 mM iodoacetamide at room temperature in the dark for 20 min. Enzymatic digestion was performed overnight in the presence of 2 µl (0.5 µg/µL) of sequencing grade trypsin (Promega, V5113) in 50 mM ammonium bicarbonate at 37 °C. Next, the addition of 2 µL of 100% trifluoracetic acid was used to terminate the digestion. Desalting was performed with C-18 ZipTip (Millipore) according to manufacturer instructions and samples were dried under nitrogen evaporator (Microvap 118, Organomation Associates, Inc) and then reconstituted with 10 µl of 0.1% Formic Acid. LC–MS/MS was conducted using an Orbitrap Fusion™ Tribrid™ Mass Spectrometer (Thermo Scientific) connected to a nanospray EASY-nLC 1200 UHPLC. Peptide mixture separation was obtained through reversed-phase chromatography using PepMap RSLC 75 μm i.d. × 15 cm long with 2 μm, C18 resin LC column (ThermoFisher). The column was first washed with mobile phase A (0.1% formic acid) and peptides were eluted at 300 nL/min using a linear gradient of 5% mobile phase B (80% acetonitrile) to 50% mobile phase B for a total of 90 min, then to 100% mobile phase B for an additional 2 min. The mass spectrometer was operated in data-dependent acquisition, each full MS scan was followed by TopN MS/MS scans in which the most intense molecular ions with charges between 2 + to 4 + were dynamically selected and fragmented by collision induced dissociation with a normalized collision energy of 35%. Acquired MS/MS spectra were searched against a fully tryptic indexed Homo sapiens database and human T-cell leukemia retrovirus database (UniProt) with Proteome Discoverer 2.1.

### Protein–protein interaction (PPIs) analysis

Search Tool for the Retrieval of Interacting Genes (STRING) was used to assess the PPI among proteins retrieved from 2 k, 10 k and 100 k EV preparations. STRING is a web-open database of known and predicted PPIs. The interactions include direct (physical) and indirect (functional) associations; they stem from computational prediction, from knowledge transfer between organisms, and from interactions aggregated from primary databases such as Biocarta, BioCyc, KEGG and Reactome. Experimental /Biochemical data comes from sources such as PDB, BioGRID, IntAct, MINT etc. The STRING database currently covers 24,584,628 proteins from 5090 organisms. For our analysis, we imported the unique proteins associated with 2 k, 10 k and 100k EVs into the STRING database; interactions with a combined score ≥ 0.70 (high confidence) were considered significant. Unique Proteins were obtained from Proteomics analysis which was performed on Mass spectrometry Data after mapping it to fully tryptic indexed Homo sapiens database (UniProt). In the PPI network, proteins are presented as nodes and edges represent the confidence level (0.7–0.9) and color represents the prediction mechanism. The confidence level ranges from 0 to 1, with 1 being the highest possible confidence.

### Cytokine measurement

Nine cytokines were screened: IL-1α, IL-18, IL-33, Calgranulin A (Calg A), granulocyte–macrophage colony-stimulating factor (GM-CSF), interferon- γ (IFN-γ), macrophage inflammatory protein-1α (MIP-1a or CCL3), MIP-1b (CCL4), and RANTES (or CCL5) in our 2 k, 10 k, and 100 k EV populations, derived from HUT102 cells, or controls (total EVs for CEM and U937) using a multiplexed bead-based assay as described previously [75]. Briefly, antibody pairs and cytokine standards were obtained from R&D Systems. Magnetic beads (Luminex) possessing 9 unique spectral areas were linked to cytokine specific capture antibodies per manufacturer’s protocols and kept at 4 °C. Lack of cross reactivity was confirmed in all cytokine pairs. An assay buffer composed of 1× PBS with 20 mM Tris–HCl, 1% each normal mouse and goat serum (Gemini Bioproducts), and 0.05% Tween 20 was used to dilute standards and samples. Additionally, standards and EV samples were also run in separate wells to determine if cytokines were membrane-bound to EVs, or with 1% Triton X to determine if cytokines were encapsulated in EVs. Bead mixtures were then added and allowed to incubate overnight at 4 °C. Triton X was combined with intact EV samples and lysed EV samples at a 1% concentration and run in different wells followed by a 3× wash of plates. Plates were then incubated with cocktails of polyclonal biotinylated anti-cytokine antibodies (R&D Systems) suspended in assay buffer for 1 h at room temperature. Plates were washed 3× prior to a 25-min incubation period with 16 μg/mL streptavidin–phycoerythrin (Invitrogen) in PBS. Afterwards, plates were washed 3× followed by resuspension of beads in PBS. A Luminex 200 analyzer with acquisition of a minimum of 100 beads for each region was used to read plates. Bioplex Manager software (BioRad) was used to analyze plates, and 5P regression algorithms were utilized for standard curves and cytokine calculations. EV surface bound cytokines were measured on intact EV samples, and EV-encapsulated cytokines were calculated as lysed EV concentrations minus EV surface concentrations.

### Angiogenesis assay

To determine the effect of EVs on tubular formation in vitro, the Angio-Ready™ Angiogenesis Assay System (ACS-2001-2™; ATCC) was used. The assay was initiated per the manufacturer’s recommended protocol. Briefly, on day 0 cells were seeded in complete Angio-Ready™ Angiogenesis Medium with VEGF (ACS-2008™; ATCC). On day 2 medium was removed and the indicated treatment was added. EV-treated cells received a 1:2 diluted formulation of the complete Angiogenesis Medium and positive control cells received complete, undiluted, Angio-Ready™ Angiogenesis Medium. EV treatments were based upon an approximate ratio of 1: 2000 (recipient cell to EV). Cells received an additional treatment on day 4. Imaging was performed using an inverted microscope fitted with the appropriate fluorescent filter.

### Flow cytometry

An mDC cell culture (1–2 × 10^6^ cells) was treated with HUT102 EVs (2 k, 10 k, and 100 k EVs), control CEM EVs (1:10,000 cell to EV), or untreated for 5 days. Cells were then fixed with 4% paraformaldehyde for 30 min on ice. Cells were then spun down and resuspended in 400 µl of cold PBS. Samples were analyzed using the BD FACS Aria II Flow Cytometer using the FACs DIVA Software. The plots show the Forward and Side scatter (FSC and SSC, respectively) data representing 10,000 events. The live mDC cell population of interest were gated and denoted by “P1”, representing 33.7% of total cells in control samples. Additionally, the dead mDC cell population was gated and denoted by “P2”, representing 49.9% of total cells in control samples. The plots of SSC and FITC fluorescent intensity are shown. EV-untreated cells were used to set baseline fluorescence levels.

### Animal model

NOD/Shi-scid/IL-2Rγ^null^ (NOG) female pregnant mice were obtained from Jackson labs. Twelve NOG pups received human CD34^+^ cells and HUT102 IRed Cells (10 Gy), as described previously [35]. Different EV populations (i.e., 2 k, 10 k, and 100 k) isolated by DUC were used to treat animals to evaluate functional effects on disease progression. Blood and Tissues from multiple organs (Spleen, Liver, L.N., and Brain) for each NOG mouse were surgically removed (0.25–0.75 cm^3^ razor) in PBS and treated for 30 min with trypsin/EDTA at 37 °C, as previously described [35]. Samples were then spun in an Eppendorf centrifuge (5415C) for 5 min at 6000 rpm. Subsequently, DNA was isolated using the Wizard® Genomic DNA Purification Kit according to the manufacturer’s protocol (Promega Corporation) and quantified via qPCR. RNA was isolated as described above and quantified via RT-qPCR. All mouse experiments were approved by the George Mason University Institutional Animal Care and Use Committee (IACUC; 0188).

### Statistical analysis

Microsoft excel was used to perform statistical analysis. Two-tailed students T-tests were used to evaluate each experiment and determine statistical significance. Sample variability was assessed to determine two-sample equal (Homoscedastic) or unequal (Heteroscedastic) variance. The degree of significance was denoted by one (*), two (**), or three asterisks (***). One asterisk was used for p-values ≤ 0.05; two asterisks for p-values ≤ 0.01; or three asterisks for p-values ≤ 0.001.

## Supplementary Information

**Additional file 1: Fig. S1.** Evaluation of average HUT102 EV sizes via NanoTracking Analysis (NTA). EVs from HUT102 cell supernatants were isolated via differential ultracentrifugation. Peak size of 2 k, 10 k, and 100 k EVs from HUT102 cells was analyzed by NTA. **Fig. S2.** Evaluation of protein cargo associated to distinct EV populations from ATL-16 cells. EVs from the HTLV-1 infected cell line, ATL-16, were isolated via differential ultracentrifugation at speeds of 2000 × *g*, 10,000 × *g*, and 100,000 × *g*, as described previously for HUT102 cells, resulting in 2 k, 10 k, and 100 k ATL-16 EVs. Proteins were evaluated via Western blot analysis for viral, cellular, and EV markers. **Fig. S3.** Validation of siRNA treatment on HTVL-1 infected cells. HTLV-1 infected cells (HUT102; Donor cells) were treated with scrambled (lane 1), CD45 (lane 2), and ICAM-1 (lane 3) siRNA to suppress translation of the target transcripts. Subsequently, EVs were isolated, as described previously, and protein content evaluated using Western blot for CD45, ICAM-1, and Actin. **Fig. S4.** RT-PCR for HTLV-1 RNA in EV Subpopulations. HTLV-1 infected cells (HUT102) were cultured and allowed to incubate for five days prior to separation of supernatant away from cells and subsequent separation of EVs into three EV subpopulations (2 k, 10 k and 100 k EVs). The viral RNA levels for *env* and *tax* were evaluated using RT-qPCR, and GAPDH as a control for cellular RNA. Statistical analyses were performed using two-tailed Student’s t test with significance indicated by “**” for p ≤ 0.01 and “***” for p ≤ 0.001.

**Additional file 2.** Mass Spectrometry of 10 k EVs isolated from HUT102 cells (HTLV-1 infected cell line) and acquired MS/MS spectra were searched against a fully tryptic indexed human T-cell leukemia retrovirus database (UniProt) with Proteome Discoverer 2.1.

## Data Availability

The data for this study is available from the corresponding author on reasonable request.

## Abbreviations

100 k EVs: EVs isolated via DUC at 100,000 × g from HUT102 supernatants
10 k EVs: EVs isolated via DUC at 10,000 × g from HUT102 supernatants
2 k EVs: EVs isolated via DUC at 2,000 × g from HUT102 supernatants
AECs: GFP expressing human aortic endothelial cells
ATLL: Adult T-cell Leukemia/Lymphoma
BBB: Blood–brain barrier
Calg A: Calgranulin A
CCF-STTG1: Astrocytes
CCs: Cellular conduits
CEM: Uninfected T-cells
CNS: Central nervous system
Control EVs: Total CEM EVs
CSF: Cerebral spinal fluid
Ct: Cycle threshold
CTLs: Cytotoxic T lymphocytes
DAMPs: Damage associated molecular patterns
DUC: Differential ultracentrifugation
ELISA: Enzyme-linked immunosorbent assay
ESCRT: Endosomal sorting complex required for transport
EVs: Extracellular vesicles
FITC: Fluorescein isothiocyanate
FN1: Fibronection-1
FSC: Forward scatter
Gly-CD63: Glycosylated CD63
GM-CSF: Granulocyte–macrophage colony-stimulating factor
HAM/TSP: HTLV-1-associated Myelopathy/Tropical Spastic Paraparesis
HTLV-1 EVs: EVs isolated from HUT102 cells
HTLV-1: Human T-cell Lymphotropic Virus Type-1
hu-NOG: Humanized NOG
IFN-I: Type-I Interferon
IFN-γ: Interferon- γ
IFN: Interferon
ILVs: Intraluminal vesicles
IR: Ionizing radiation
L.N.: Lymph node
LCMS/MS: Liquid chromatography tandem mass spectrometry
MBV: Multi-vesicular body
MDM: Monocytic cell (THP-1 or U937)-derived macrophages
mDCs: Monocytic cell (THP-1)-derived dendritic cells
MIP-1a: Macrophage inflammatory protein-1a
MSCs: Mesenchymal stem cells
NOG: NOD/Shi-scid/IL-2Rγnull
NTA: Nanoparticle tracking analysis
PAMPs: Pathogen associated molecular patterns
PBMCs: Peripheral blood mononuclear cells
PBS: Phosphate Buffered Saline
pDCs: Plasmacytoid dendritic cells
PPI: Protein–protein interaction
PRRs: Pattern recognition receptors
RANTES: Regulated on activation normally T-cell expressed and secreted
siRNA: Small interfering RNA
SSC: Side-scatter
TNTs: Tunneling nanotubes
Total EVs: 2 k, 10 k, and 100 k HTLV-1 EVs
U937: Uninfected myeloid cell line
VB: Viral biofilm
VS: Virological synapse
