# Supplementary material for: Extracellular vesicles from HTLV-1 infected cells modulate target cells and viral spread
Source: Retrovirology. 2021 Feb 23;18:6. doi: 10.1186/s12977-021-00550-8 (PMC7901226; doi:10.1186/s12977-021-00550-8)
Supplement: Supplementary file 1 — Additional file 1: Fig. S1. Evaluation of average HUT102 EV sizes via NanoTracking Analysis (NTA). EVs from HUT102 cell supernatants were isolated via differential ultracentrifugation. Peak size of 2 k, 10 k, and 100 k EVs from HUT102 cells was analyzed by NTA. Fig. S2. Evaluation of protein cargo associated to distinct EV populations from ATL-16 cells. EVs from the HTLV-1 infected cell line, ATL-16, were isolated via differential ultracentrifugation at speeds of 2000 × g, 10,000 × g, and 100,000 × g, as described previously for HUT102 cells, resulting in 2 k, 10 k, and 100 k ATL-16 EVs. Proteins were evaluated via Western blot analysis for viral, cellular, and EV markers. Fig. S3. Validation of siRNA treatment on HTVL-1 infected cells. HTLV-1 infected cells (HUT102; Donor cells) were treated with scrambled (lane 1), CD45 (lane 2), and ICAM-1 (lane 3) siRNA to suppress translation of the target transcripts. Subsequently, EVs were isolated, as described previously, and protein content evaluated using Western blot for CD45, ICAM-1, and Actin. Fig. S4. RT-PCR for HTLV-1 RNA in EV Subpopulations. HTLV-1 infected cells (HUT102) were cultured and allowed to incubate for five days prior to separation of supernatant away from cells and subsequent separation of EVs into three EV subpopulations (2 k, 10 k and 100 k EVs). The viral RNA levels for env and tax were evaluated using RT-qPCR, and GAPDH as a control for cellular RNA. Statistical analyses were performed using two-tailed Student’s t test with significance indicated by “**” for p ≤ 0.01 and “***” for p ≤ 0.001. [file 12977_2021_550_MOESM1_ESM.pdf]

Additional file 1

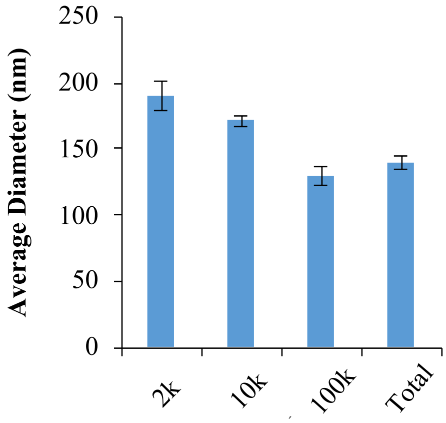

Fig. S1. Evaluation of average HUT102 EV sizes via NanoTracking Analysis (NTA). EVs from HUT102 cell supernatants were isolated via differential ultracentrifugation. Peak size of 2k, 10k, and 100k EVs from HUT102 cells was analyzed by NTA.

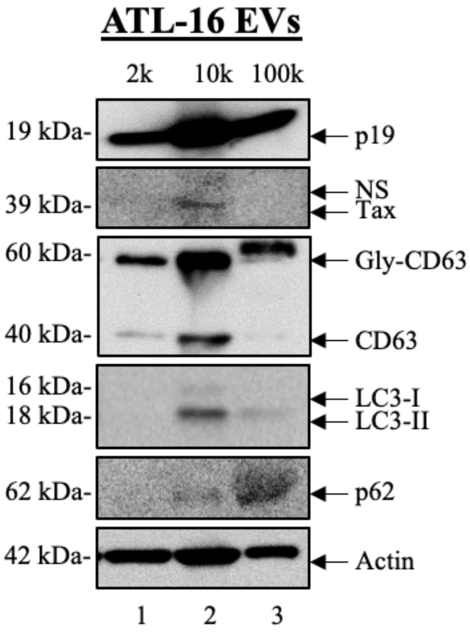

Fig. S2. Evaluation of protein cargo associated to distinct EV populations from ATL-16 cells. EVs from the HTLV-1 infected cell line, ATL-16, were isolated via differential ultracentrifugation at speeds of 2,000 x *g*, 10,000 x *g*, and 100,000 x *g*, as described previously for HUT102 cells, resulting in 2k, 10k, and 100k ATL-16 EVs. Proteins were evaluated via Western blot analysis for viral, cellular, and EV markers.

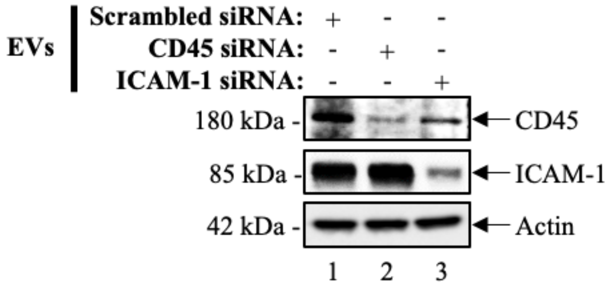

Fig. S3. Validation of siRNA treatment on HTVL-1 infected cells. HTLV-1 infected cells (HUT102; Donor cells) were treated with scrambled (lane 1), CD45 (lane 2), and ICAM-1 (lane 3) siRNA to suppress translation of the target transcripts. Subsequently, EVs were isolated, as described previously, and protein content evaluated using Western blot for CD45, ICAM-1, and Actin.

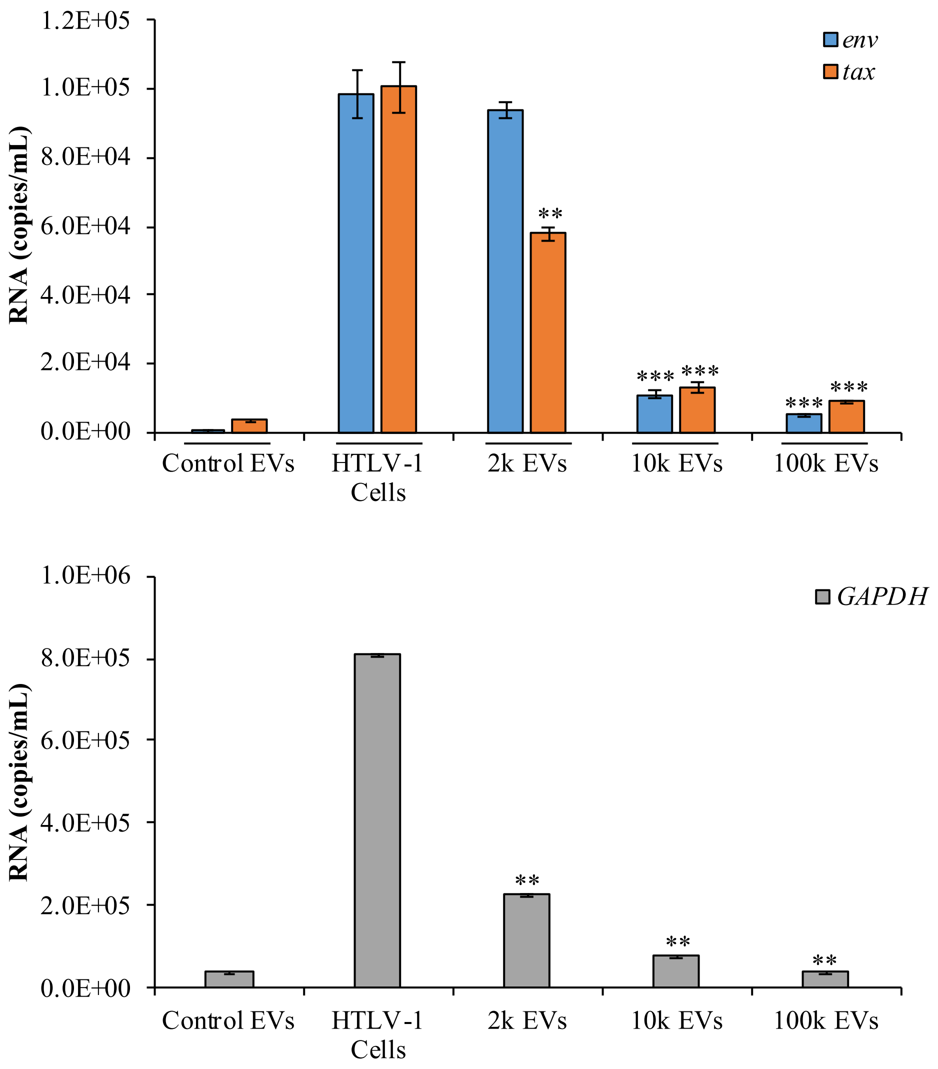

Fig. S4. RT-PCR for HTLV-1 RNA in EV Subpopulations. HTLV-1 infected cells (HUT102) were cultured and allowed to incubate for five days prior to separation of supernatant away from cells and subsequent separation of EVs into three EV subpopulations (2k, 10k and 100k EVs). The viral RNA levels for env and tax were evaluated using RT-qPCR, and GAPDH as a control for cellular RNA. Statistical analyses were performed using two-tailed Student's t test with significance indicated by "**" for $p \leq 0.01$ and "***" for $p \leq 0.001$.
